# FXR inhibition may protect from SARS-CoV-2 infection by reducing ACE2

**DOI:** 10.1038/s41586-022-05594-0

**Published:** 2022-12-05

**Authors:** Teresa Brevini, Mailis Maes, Gwilym J. Webb, Binu V. John, Claudia D. Fuchs, Gustav Buescher, Lu Wang, Chelsea Griffiths, Marnie L. Brown, William E. Scott, Pehuén Pereyra-Gerber, William T. H. Gelson, Stephanie Brown, Scott Dillon, Daniele Muraro, Jo Sharp, Megan Neary, Helen Box, Lee Tatham, James Stewart, Paul Curley, Henry Pertinez, Sally Forrest, Petra Mlcochova, Sagar S. Varankar, Mahnaz Darvish-Damavandi, Victoria L. Mulcahy, Rhoda E. Kuc, Thomas L. Williams, James A. Heslop, Davide Rossetti, Olivia C. Tysoe, Vasileios Galanakis, Marta Vila-Gonzalez, Thomas W. M. Crozier, Johannes Bargehr, Sanjay Sinha, Sara S. Upponi, Corrina Fear, Lisa Swift, Kourosh Saeb-Parsy, Susan E. Davies, Axel Wester, Hannes Hagström, Espen Melum, Darran Clements, Peter Humphreys, Jo Herriott, Edyta Kijak, Helen Cox, Chloe Bramwell, Anthony Valentijn, Christopher J. R. Illingworth, UK-PBC Consortium, Bassam Dahman, Dustin R. Bastaich, Raphaella D. Ferreira, Thomas Marjot, Eleanor Barnes, Andrew M. Moon, Alfred S. Barritt, Ravindra K. Gupta, Stephen Baker, Anthony P. Davenport, Gareth Corbett, Vassilis G. Gorgoulis, Simon J. A. Buczacki, Joo-Hyeon Lee, Nicholas J. Matheson, Michael Trauner, Andrew J. Fisher, Paul Gibbs, Andrew J. Butler, Christopher J. E. Watson, George F. Mells, Gordon Dougan, Andrew Owen, Ansgar W. Lohse, Ludovic Vallier, Fotios Sampaziotis

**Affiliations:** 1 Wellcome–MRC Cambridge Stem Cell Institute, Cambridge, UK; 2 Cambridge Institute of Therapeutic Immunology & Infectious Disease (CITIID), Department of Medicine, University of Cambridge, Cambridge, UK; 3 Cambridge Liver Unit, Cambridge University Hospitals NHS Foundation Trust, Cambridge, UK; 4 Division of Gastroenterology and Hepatology, University of Miami and Miami VA Health System, Miami, FL USA; 5 Hans Popper Laboratory of Molecular Hepatology, Division of Gastroenterology and Hepatology, Department of Internal Medicine III, Medical University of Vienna, Vienna, Austria; 6 Department of Medicine, University Medical Centre Hamburg-Eppendorf, Hamburg, Germany; 7 Transplant and Regenerative Medicine Laboratory, Translational and Clinical Research Institute, Faculty of Medical Sciences, Newcastle University, Newcastle upon Tyne, UK; 8 Department of Medicine, University of Cambridge, Cambridge, UK; 9 Wellcome Sanger Institute, Hinxton, UK; 10 Centre of Excellence in Long-acting Therapeutics (CELT), Department of Pharmacology and Therapeutics, Institute of Systems, Molecular and Integrative Biology, University of Liverpool, Liverpool, UK; 11 Department of Infection Biology and Microbiomes, Institute of Infection, Veterinary and Ecological Sciences, University of Liverpool, Liverpool, UK; 12 Nuffield Department of Surgical Sciences, University of Oxford, Oxford, UK; 13 Academic Department of Medical Genetics, University of Cambridge, Cambridge, UK; 14 Experimental Medicine and Immunotherapeutics, University of Cambridge, Addenbrooke’s Hospital, Cambridge, UK; 15 Department of Surgery, University of Cambridge and NIHR Cambridge Biomedical Research Centre, Cambridge, UK; 16 Division of Cardiovascular Medicine, University of Cambridge, Cambridge, UK; 17 Department of Radiology, Cambridge University Hospitals NHS Foundation Trust, Cambridge, UK; 18 Roy Calne Transplant Unit, Cambridge University Hospitals NHS Foundation Trust, Cambridge, UK; 19 Department of Histopathology, Cambridge University Hospitals NHS Foundation Trust, Cambridge, UK; 20 Department of Medicine, Huddinge, Karolinska Institutet, Stockholm, Sweden; 21 Norwegian PSC Research Center, Department of Transplantation Medicine, Division of Surgery, Inflammatory Diseases and Transplantation, Oslo University Hospital, Rikshospitalet, Oslo, Norway; 22 Research Institute of Internal Medicine, Division of Surgery, Inflammatory Diseases and Transplantation, Oslo University Hospital, Rikshospitalet, Oslo, Norway; 23 Institute of Clinical Medicine, Faculty of Medicine, University of Oslo, Oslo, Norway; 24 Section of Gastroenterology, Department of Transplantation Medicine, Division of Surgery, Inflammatory Diseases and Transplantation, Oslo University Hospital, Rikshospitalet, Oslo, Norway; 25 Hybrid Technology Hub Centre of Excellence, Institute of Basic Medical Sciences, Faculty of Medicine, University of Oslo, Oslo, Norway; 26 MRC–University of Glasgow Centre for Virus Research, Glasgow, UK; 27 Department of Applied Mathematics and Theoretical Physics, University of Cambridge, Cambridge, UK; 28 Department of Health Behavior and Policy, Virginia Commonwealth University, Richmond, VA USA; 29 Oxford Liver Unit, Translational Gastroenterology Unit, Oxford University Hospitals NHS Foundation Trust, University of Oxford, Oxford, UK; 30 Division of Gastroenterology and Hepatology, University of North Carolina, Chapel Hill, NC USA; 31 Cambridge University Hospitals NHS Foundation Trust, Cambridge, UK; 32 Department of Histology and Embryology, School of Medicine, National and Kapodistrian University of Athens, Athens, Greece; 33 Ninewells Hospital and Medical School, University of Dundee, Dundee, UK; 34 Biomedical Research Foundation, Academy of Athens, Athens, Greece; 35 Department of Physiology, Development and Neuroscience, University of Cambridge, Cambridge, UK; 36 NHS Blood and Transplant, Cambridge, UK; 37 National Institute of Health Research (NIHR) Cambridge Biomedical Research Centre, and the NIHR Blood and Transplant Research Unit (BTRU) at the University of Cambridge in collaboration with Newcastle University and in partnership with NHS Blood and Transplant (NHSBT), Cambridge, UK; 38 Berlin Institute of Health (BIH), BIH Centre for Regenerative Therapies (BCRT), Charité—Universitätsmedizin Berlin, Berlin, Germany; 39 Max Planck Institute for Molecular Genetics, Berlin, Germany

**Keywords:** Translational research, Preclinical research, SARS-CoV-2, Adjuvants, Viral infection

## Abstract

Preventing SARS-CoV-2 infection by modulating viral host receptors, such as angiotensin-converting enzyme 2 (ACE2)^1^, could represent a new chemoprophylactic approach for COVID-19 that complements vaccination^2,3^. However, the mechanisms that control the expression of ACE2 remain unclear. Here we show that the farnesoid X receptor (FXR) is a direct regulator of *ACE2* transcription in several tissues affected by COVID-19, including the gastrointestinal and respiratory systems. We then use the over-the-counter compound z-guggulsterone and the off-patent drug ursodeoxycholic acid (UDCA) to reduce FXR signalling and downregulate ACE2 in human lung, cholangiocyte and intestinal organoids and in the corresponding tissues in mice and hamsters. We show that the UDCA-mediated downregulation of ACE2 reduces susceptibility to SARS-CoV-2 infection in vitro, in vivo and in human lungs and livers perfused ex situ. Furthermore, we reveal that UDCA reduces the expression of ACE2 in the nasal epithelium in humans. Finally, we identify a correlation between UDCA treatment and positive clinical outcomes after SARS-CoV-2 infection using retrospective registry data, and confirm these findings in an independent validation cohort of recipients of liver transplants. In conclusion, we show that FXR has a role in controlling ACE2 expression and provide evidence that modulation of this pathway could be beneficial for reducing SARS-CoV-2 infection, paving the way for future clinical trials.

## Abstract (web summary)

FXR regulates the levels of ACE2 in tissues of the respiratory and gastrointestinal systems that are affected by COVID-19, and inhibiting FXR with ursodeoxycholic acid downregulates ACE2 and reduces susceptibility to SARS-CoV-2 infection.

## Main

Since the beginning of the pandemic, the management of COVID-19 has improved considerably with the development of therapeutic agents, vaccines and monoclonal antibodies^4^. Despite the transformational effect of vaccines in populations that can access them, major global health challenges remain. New SARS-CoV-2 variants continue to emerge and are associated with high case rates and substantial global mortality. Treatment options, such as dexamethasone, remdesivir, molnupiravir and nirmatrelvir, improve clinical outcomes only in specific groups of patients^5,6^; monoclonal antibodies, such as the REGN-COV2 cocktail, show reduced neutralizing efficacy against new variants^7^; and vaccines are restricted by variable efficacy^8^, the emergence of vaccine-resistant viral variants^7^, cost^9^ and availability^10^. Finally, one of the biggest challenges is still prophylaxis in vulnerable and high-risk groups, such as immunocompromised individuals who are not expected to mount an appropriate response to vaccines. The only prophylactic agents for these groups are monoclonal antibodies, which are hampered by the propensity of the viral spike to evolve to escape neutralization^7^. Notably, there are at present no other approved agents for pharmacological prophylaxis against COVID-19^2^. Therefore, there is a pressing need for novel prophylactic agents that reduce the risk of severe disease^3^, are less susceptible to viral resistance and are compatible with healthcare systems in low- and middle-income countries.

Viral host receptors represent logical therapeutic targets, because they are essential for SARS-CoV-2 cellular entry and infection^1^. Among these, ACE2 is particularly appealing^1^. ACE2 is a transmembrane carboxypeptidase with a broad substrate specificity, including angiotensin II, that acts as the main receptor for SARS-CoV-2. It directly binds to the spike proteins of different coronaviruses, with a high affinity for SARS-CoV-2, rendering it indispensable for viral entry^11^. Accordingly, COVID-19 predominantly affects tissues that express ACE2, such as the lungs, the cardiovascular system, the digestive tract and the biliary tree^12,13^.

Modifying the expression of ACE2 could impede viral entry and protect against infection with SARS-CoV-2 and potentially other coronaviruses that use the same receptor. Furthermore, because ACE2 is a host-cell protein, its expression is not likely to be affected by mutations in the virus. Therefore, therapies that modulate ACE2 expression may be effective against multiple SARS-CoV-2 variants with a higher genetic barrier to resistance. However, the mechanisms that control ACE2 expression remain unclear. Here we use human cholangiocyte organoids as a proof-of-principle system to demonstrate that the bile acid receptor FXR controls the expression of ACE2. We show that this mechanism applies in several SARS-CoV-2-affected tissues, including gastrointestinal and respiratory epithelia. Subsequently, we demonstrate that suppressing FXR signalling, by using the approved drug UDCA or the over-the-counter phytosteroid z-guggulsterone (ZGG), reduces ACE2 expression and SARS-CoV-2 infection in vitro and in an airborne transmission model in golden Syrian hamsters. We repeat our experiments in human lungs and livers perfused ex situ and show that administering UDCA at physiologically relevant concentrations reduces ACE2 and viral infection in both organs ex vivo. We then demonstrate a reduction in the levels of ACE2 in the nasal epithelium of volunteers receiving clinically approved doses of UDCA. Finally, we interrogate an international registry cohort of patients with COVID-19 and chronic liver disease, identify a correlation between UDCA therapy and better clinical outcomes from COVID-19 and reproduce these results in a second independent cohort of liver-transplant recipients.

## Bile acids modulate cholangiocyte ACE2

**Extended Data Fig. 1:**
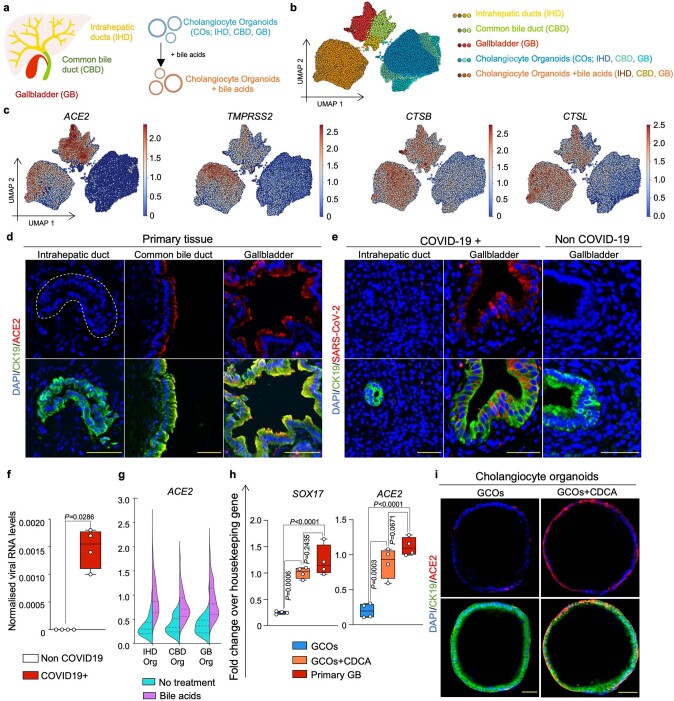
Expression of SARS-CoV-2 entry genes in cholangiocytes. (**a**) Schematic illustration of different primary human cholangiocyte populations corresponding to different areas of the biliary tree and COs derived from different areas of the biliary tree grown in absence or presence of the bile acids. (**b**) UMAP plot illustrating different cholangiocyte populations from (a) analysed by scRNA-seq. (**c**) UMAP plots showing that viral entry related genes are predominantly expressed in extrahepatic cholangiocytes and COs treated with bile acids. (**d–e**) Immunofluorescence illustrating that ACE2 is expressed in extrahepatic cholangiocytes (**d**) and that SARS-CoV-2 infects gall bladder cholangiocytes of patients with COVID-19 but not intrahepatic cholangiocytes (**e**). N = 4 independent samples. Scale bars 50 μm. (**f**) QPCR confirming detection of SARS-CoV-2 RNA in bile of patients with COVID-19. Housekeeping gene, HMBS; n = 4; two-tailed Mann–Whitney test; centre line, median; box, interquartile range (IQR); whiskers, range; bars, standard deviation. (**g**) Violin plot of scRNA-seq data from (b) showing that COs upregulate ACE2 when treated with bile acids regardless of their region of origin. (**h**) QPCR validating that upon treatment with the bile acid CDCA COs assume a gall bladder identity expressing the gall bladder marker SOX17 and upregulating ACE2 at levels comparable to primary gall bladder. Housekeeping gene, HMBS; n = 4 independent experiments; one-way ANOVA adjusted for multiple comparisons; ns, non-significant; centre line, median; box, interquartile range (IQR); whiskers, range; bars, standard deviation. (**i**) Immunofluorescence showing that CDCA induces ACE2 expression in GCOs. N = 4 independent experiments. Scale bars 50 μm.

**Extended Data Fig. 2:**
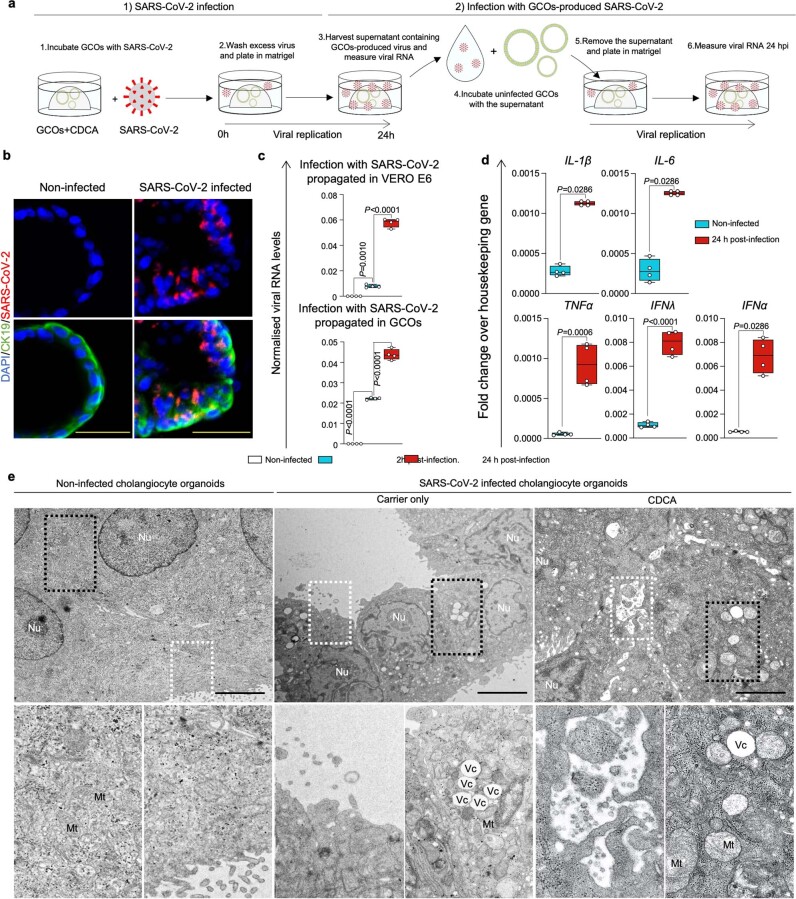
CDCA-treated GCOs can be infected with SARS-CoV-2. (**a**) Schematic representation of the methodology used to infect GCOs with SARS-CoV-2 and test the capacity of SARS-CoV-2 virions produced in GCOs to infect new (uninfected) cells. (**b**) Immunofluorescence validating SARS-CoV-2 infection in GCOs. N = 4 independent experiments. Scale bars 50 μm. (**c**) QPCR confirming infection of CDCA-treated GCOs with SARS-CoV-2 propagated in VERO E6 cells (top panel) and with SARS-CoV-2 propagated in GCOs treated with CDCA (bottom panel), illustrating that SARS-CoV-2 produced in GCOs+CDCA retains its infectious capacity. Scale bars 50 μm; housekeeping gene, GAPDH; n = 4 independent experiments; Kruskal–Wallis test adjusted for multiple comparisons; centre line, median; box, interquartile range (IQR); whiskers, range; bars, standard deviation. (**d**) QPCR showing upregulation of innate immune and antiviral response genes in GCOs+CDCA following SARS-CoV-2 infection. Housekeeping gene, GAPDH; n = 4, 2 biological and 2 technical replicates; two-tailed Mann–Whitney test (IL-1β, IL-6, IFNα) and two-tailed unpaired t-test (TNF, IFNλ). (CDCA concentration, 10 μM); centre line, median; box, interquartile range (IQR); whiskers, range; bars, standard deviation. (**e**) Transmission electron micrograph of uninfected COs (left panel) and COs infected with SARS-CoV-2 in the absence (central panel) or presence of CDCA (right panel) showing key morphological features of viral infection and cell death, such as production of viral particles, formation of pathologic vacuoles (Vc) and swollen mitochondria (Mt). N = 3 independent experiments. Scale bars 5 μm.

To investigate the mechanisms that control ACE2 expression, we used cholangiocyte organoids as a proof-of-principle model. Cholangiocytes are epithelial cells that line the lumen of the bile ducts and the gall bladder. We decided to focus on gall bladder cholangiocytes for several reasons. Cholangiocytes of the gall bladder express the highest levels of ACE2 in the biliary tree^12^ (Wilcoxon rank-sum test *P* < 4.5^−201^) (Extended Data Fig. 1a–d) and one of the highest levels of ACE2 in the body^12^. Thus, they can be infected by SARS-CoV-2 (Extended Data Fig. 1e,f). Furthermore, they can be propagated as organoids^14–16^ (Extended Data Fig. 1a–c) and maintain their gall bladder identity in vitro (Extended Data Fig. 1a–c) after the bile acid chenodeoxycholic acid (CDCA) is added to their culture medium^14^. The resulting gall bladder cholangiocyte organoids (GCOs) express high levels of ACE2 (Extended Data Fig. 1c,g–i), retain their capacity to be infected by SARS-CoV-2 (Extended Data Fig. 2a–e), produce infective viral progeny (Extended Data Fig. 2c) and appropriately upregulate the expression of innate immune genes and antiviral response markers (Extended Data Fig. 2d). Of note, in the absence of bile acids (CDCA), cholangiocyte organoids lose the expression of gall bladder markers, including ACE2 (Extended Data Fig. 1g–i), which shows that CDCA is required for the expression of ACE2. These results not only show that GCOs provide an appropriate platform through which to study the mechanisms that control the expression of ACE2 in human cells, but also identify CDCA as a key regulator of SARS-CoV-2 receptor levels.

## Bile acids control ACE2 levels through FXR

**Fig. 1:**
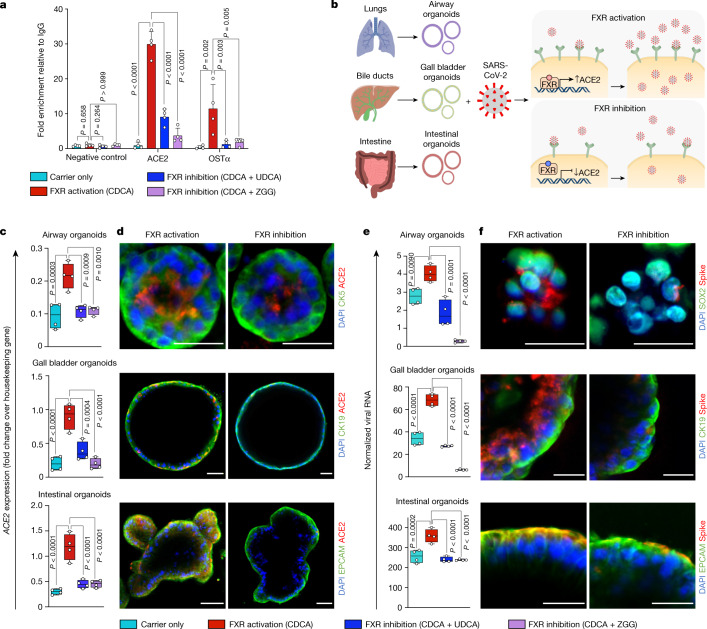
FXR modulates ACE2 expression and SARS-CoV-2 infection. **a**, ChIP–qPCR on cholangiocyte organoids, showing that the FXR agonist CDCA promotes the binding of FXR on the *ACE2* promoter, and that this is reduced by FXR inhibitors (UDCA and ZGG). OSTα as positive control; *ACE2* promoter adjoining region as negative control; *n* = 4 independent experiments; one-way ANOVA adjusted for multiple comparisons; bars,s.d. **b**, Schematic representation of the suggested mechanism for FXR-mediated control of ACE2 expression and SARS-CoV-2 infection relative to **e**,**f**. **c**,**d**, qPCR (**c**) and immunofluorescence (**d**) showing the levels of *ACE2* after modulation of FXR activity in primary airway, biliary and intestinal organoids. Housekeeping gene, *HMBS* (also known as *PBGD*); *n* = 4 independent experiments; one-way ANOVA; centre line, median; box, interquartile range (IQR); whiskers, range; bars, s.d. Yellow scale bars, 50 μm; grey scale bars, 25 μm. **e**, qPCR quantifying SARS-CoV-2 viral RNA 24 h after infection in primary organoids treated with physiological levels of bile acids (CDCA) in the presence or absence of FXR inhibitors (UDCA and ZGG). Housekeeping gene, *GAPDH*; *n* = 4 independent experiments; one-way ANOVA adjusted for multiple comparisons; centre line, median; box, interquartile range (IQR); whiskers, range; bars, s.d. **f**, Immunofluorescence images showing the presence of SARS-CoV-2 spike protein 24 hours after infection in organoids corresponding to **e**. Scale bars, 25 μm. CDCA, UDCA and ZGG concentration, 10 μM.

**Extended Data Fig. 3:**
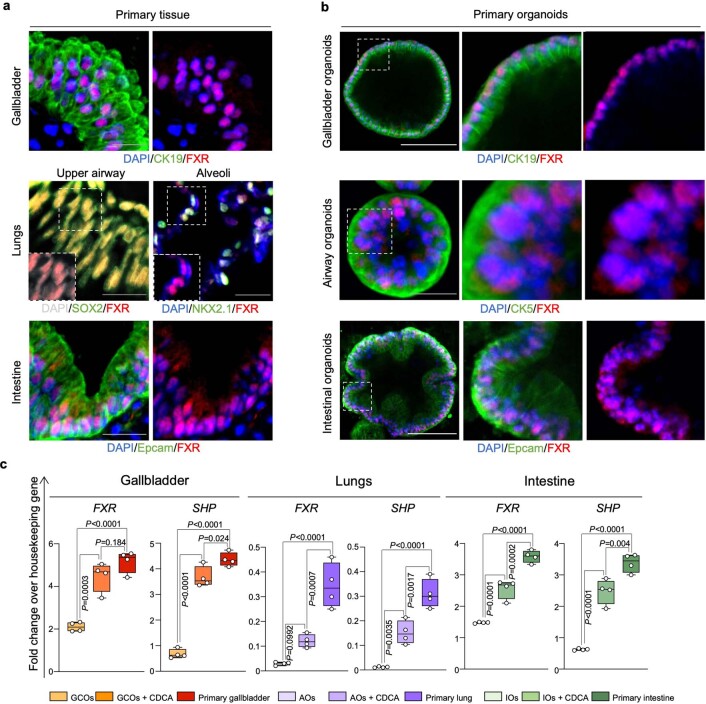
FXR is present and active in tissues affected by COVID-19 and their corresponding CDCA-treated organoids. (**a–b**) Immunofluorescence images confirming expression of FXR in primary human gall bladder, lungs and intestinal tissue (**a**) and in corresponding primary organoids treated with physiological levels of bile acids (CDCA, 10 μM) (**b**). N = 4 independent experiments. White scale bars 100 μm; grey scale bar 25 μm. (**c**) QPCR analysis validating expression of FXR and its downstream effector SHP in primary tissue and corresponding organoids in presence or absence of physiological levels of bile acids (CDCA). CDCA treatment increases FXR and SHP expression in organoids to levels that are closer to primary tissue. Housekeeping gene, HMBS; n = 4 independent experiments; one-way ANOVA adjusted for multiple comparisons; centre line, median; box, interquartile range (IQR); whiskers, range; bars, standard deviation. AOs, airway organoids; IOs, intestinal organoids.

**Extended Data Fig. 4:**
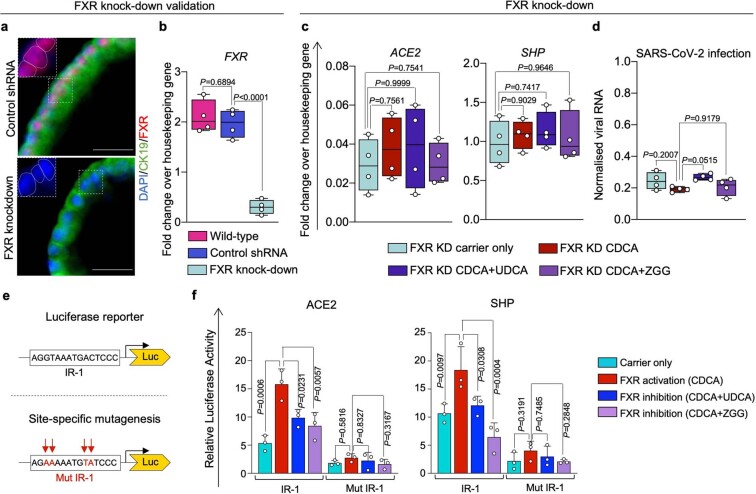
UDCA and ZGG require FXR to reduce ACE2 and SARS-CoV-2 infection. (**a–b**) Immunofluorescence (**a**) and QPCR (**b**) showing downregulation of FXR expression following FXR knockdown (KD) in COs. Scale bars 25 μm. Housekeeping gene HMBS. One-way ANOVA adjusted for multiple comparisons; n = 4 independent experiments; centre line, median; box, interquartile range; whiskers, range; bars, standard deviation. (**c**) QPCR on FXR KD COs showing no change in the expression of ACE2 and the FXR downstream effector SHP following treatment with CDCA, UDCA or ZGG, demonstrating that FXR is indispensable for regulating ACE2 and SHP through these compounds. ACE2 and SHP expression in wild-type organoids shown in Fig. 1c and Extended Data Fig. 5a. Housekeeping gene, HMBS; n = 4; one-way ANOVA adjusted for multiple comparisons; ns, non-significant; centre line, median; box, interquartile range; whiskers, range; bars, standard deviation. (**d**) QPCR quantifying SARS-CoV-2 RNA 24 h post infection in FXR KD COs treated with CDCA, UDCA or ZGG. SARS-CoV-2 infection in the presence of FXR is shown in Fig. 1e. Housekeeping gene, GAPDH; n = 4 independent experiments; one-way ANOVA adjusted for multiple comparisons; centre line, median; box, interquartile range; whiskers, range; bars, standard deviation. (CDCA, UDCA and ZGG concentration, 10 μM). (**e**) Schematic illustrating the luciferase reporter construct containing the FXRE IR-1 identified in Fig. 1a and the mutagenesis strategy used in panel (f). (**f**) Luciferase reporter assay in HEK293 cells showing the transcriptional activity associated with the IR-1 located in the ACE2 promoter upon treatment with CDCA, UDCA or ZGG. Site-directed mutagenesis on IR-1 abolishes FXR binding/transactivation confirming the specificity of FXR binding on the ACE2 IR-1. IR-1 located in the SHP promoter used as positive control. N = 3 independent experiments; one-way ANOVA adjusted for multiple comparisons; bars, standard deviations. (CDCA, UDCA and ZGG concentration, 50 μM).

**Extended Data Fig. 5:**
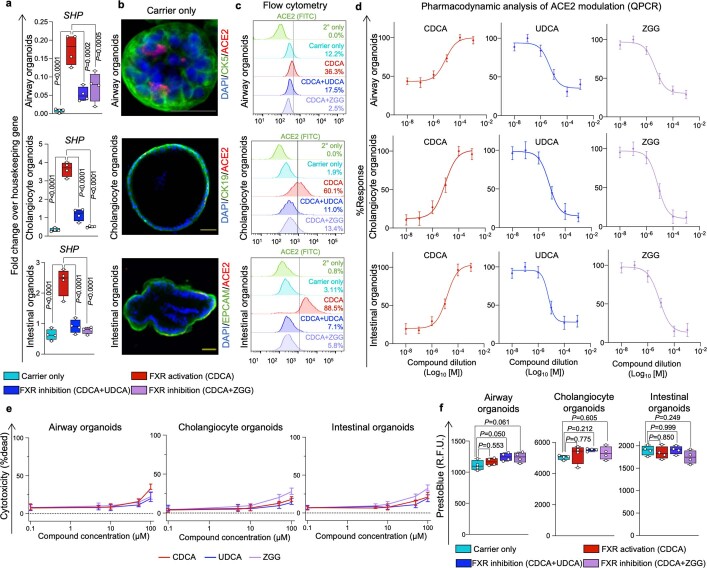
FXR modulation in biliary, airway and intestinal organoids. (**a**) QPCR analysis in primary airway, biliary and intestinal organoids demonstrating that CDCA activates FXR, whereas UDCA and ZGG inhibit it, as evidenced by corresponding changes in the expression of the FXR downstream target SHP. Housekeeping gene, HMBS; n = 4 independent experiments; one-way ANOVA adjusted for multiple comparisons; centre line, median; box, interquartile range (IQR); whiskers, range; bars, standard deviation. (**b**) Immunofluorescence showing ACE2 expression levels in primary organoids in absence of bile acids. The panel is complementary to Fig. 1d showing the modulation of ACE2 following FXR activation (CDCA) and inhibition (UDCA/ZGG). N = 4 independent experiments. Yellow scale bars 50 μm; grey scale bar 25 μm. (**c**) Flow cytometry histograms showing changes in ACE2 levels upon modulation of FXR activity in primary airway, biliary and intestinal organoids. n = 3 independent experiments. (CDCA, UDCA and ZGG concentration, 10 μM). (**d**) Dose–response curves showing the effect of 0.01 μM – 1 mM of CDCA, UDCA and ZGG on the expression of ACE2 in primary airway, biliary and intestinal organoids (n = 3 independent experiments). Response defined as percentage of the maximal ACE2 expression level for each condition via QPCR. Bars, SEM. (**e**) Percentage of non-viable cells following treatment of airway, biliary and intestinal organoids with CDCA, UDCA and ZGG at a range of 0.1 μM – 100 μM showing that these compounds cause minimal cell death within the tested range. N = 3 independent experiments. Bars, range. (**f**) Resazurin assay (PrestoBlue) showing that treatment with 10 μM of CDCA, UDCA or ZGG does not affect cellular viability. N = 4 independent experiments; one-way ANOVA adjusted for multiple comparisons; ns, non-significant; centre line, median; box, interquartile range (IQR); whiskers, range; bars, standard deviation.

Because the bile acid CDCA is the most potent natural agonist of the bile acid receptor and transcription factor FXR^17^, we hypothesized that CDCA could act through FXR to control the expression of ACE2. To test this hypothesis, we first confirmed that FXR is expressed in gall bladder cholangiocytes in vivo and in the corresponding GCOs in vitro (Extended Data Fig. 3a–c) and that it is activated by treatment with CDCA, as evidenced by the upregulation of its downstream target small heterodimer partner (SHP) (Extended Data Fig. 3c). To confirm that FXR is essential for the CDCA-induced upregulation of ACE2, we knocked down the expression of FXR in cholangiocyte organoids by using short hairpin RNAs (shRNAs) and found that this prevented the upregulation of ACE2 and SHP after treatment with CDCA (Extended Data Fig. 4a–c). To assess whether FXR could bind to *ACE2* and potentially control its transcriptional activity, we analysed the *ACE2* promoter region and identified the presence of the FXR response element (FXRE) IR-1. Accordingly, we used chromatin immunoprecipitation followed by quantitative PCR (ChIP–qPCR) to confirm that activated FXR directly binds to the *ACE2* promoter (Fig. 1a), and showed the functional relevance of this binding using a luciferase reporter that contains the IR-1 region of *ACE2* (Extended Data Fig. 4e,f). Site-specific mutagenesis on the IR-1 region reduced the luciferase signal (Extended Data Fig. 4e,f), demonstrating the specificity of the FXR-binding site on the *ACE2* promoter. Conversely, suppression of FXR signalling, using the FXR antagonist ZGG^18^ or the clinically used drug UDCA^17^, reduced the activity of FXR (as evidenced by decreased levels of SHP; Extended Data Fig. 5a), reduced the presence of FXR on the *ACE2* promoter (Fig. 1a and Extended Data Fig. 4e,f) and downregulated the expression of ACE2 at the transcript and protein levels (Fig. 1c,d and Extended Data Fig. 5b–d). Considered together, these results show that FXR directly controls the expression of ACE2 in cholangiocytes (Fig. 1b).

## FXR regulates ACE2 in various cell types

FXR is expressed in several cell types^17,19–21^ and can be activated by bile acids, which are present not only in the gastrointestinal tract^22^ but also in the lungs^20,21^ and in the systemic circulation^22^. Thus, ACE2 regulation through FXR may represent a general mechanism, extending beyond cholangiocytes. To examine this possibility, we repeated our experiments using primary organoids from key organs infected by SARS-CoV-2^23^, such as the lungs and the intestine. The relevance of these platforms for studying SARS-CoV-2 infection has already been shown^24,25^. We first confirmed the expression of FXR in these tissues, both in vivo and in vitro (Extended Data Fig. 3a–c). Subsequently, we showed that treatment with physiological concentrations of CDCA (10 μM)^22^ resulted in the activation of FXR, as evidenced by the upregulation of the FXR downstream target SHP (Extended Data Fig. 3c) and by increased ACE2 expression (Fig. 1c,d and Extended Data Fig. 5a–c). Conversely, suppression of FXR signalling by UDCA or ZGG reduced the levels of ACE2 and SHP in primary airway and intestinal organoids (Fig. 1c,d and Extended Data Fig. 5a–d). Notably, CDCA, UDCA and ZGG had no cytotoxic effects in the concentration range that was used for our experiments (Extended Data Fig. 5e,f). These results confirm that FXR participates in the regulation of ACE2 expression in organoids derived from the respiratory, biliary and intestinal epithelium, suggesting that FXR-mediated control of ACE2 expression may be relevant for several organs (Fig. 1b).

## FXR regulates viral infection in vitro

**Extended Data Fig. 6:**
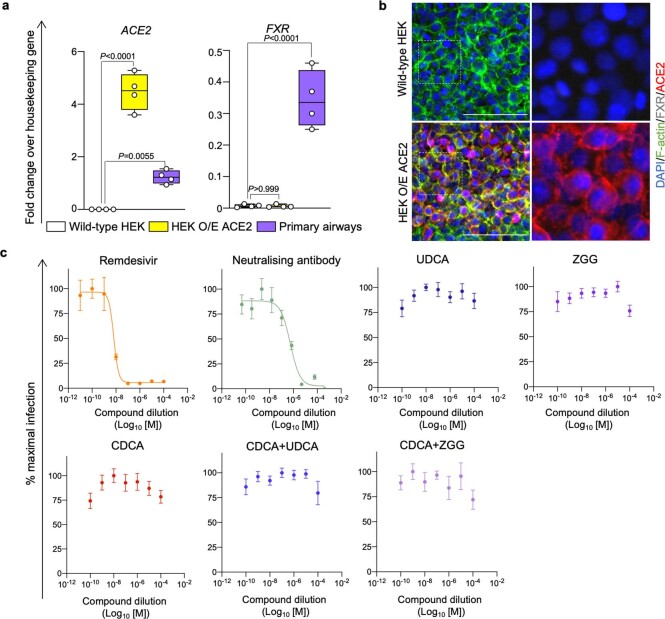
ACE2 downregulation is required for the UDCA- or ZGG-mediated reduction in SARS-CoV-2 infection. (**a**-**b**) QPCR analysis (**a**) and immunofluorescence (**b**) illustrating ACE2 and FXR expression in wild-type HEK293 cells and HEK293T cells stably expressing ACE2. Primary human airway tissue used as positive control. Housekeeping gene, HMBS; n = 4; one-way ANOVA adjusted for multiple comparisons; centre line, median; box, interquartile range (IQR); whiskers, range; bars, standard deviation. Scale bars 100 μm. (**c**) SARS-CoV-2 infection of HEK293T cells genetically engineered to stably express ACE2. Cells were treated with the indicated doses of CDCA, UDCA or ZGG, infected with SARS-CoV-2 at an MOI of 0.01, and analysed after 24 h. The SARS-CoV-2 RdRp inhibitor remdesivir and a neutralizing antibody cocktail blocking the interaction between SARS-CoV-2 spike and ACE2 (REGN-COV2) were included as positive controls. N = 3; one-way ANOVA adjusted for multiple comparisons; mean values ± SEM.

Our results show that suppressing FXR signalling with the clinically approved drug UDCA—which is used as a first-line treatment in primary biliary cholangitis (PBC)^26^—or with the over-the-counter drug ZGG reduces the expression of ACE2 in multiple cell types. To consider the relevance of this finding for COVID-19, we investigated whether the FXR-mediated downregulation of ACE2 could reduce susceptibility to SARS-CoV-2 infection in vitro. For this, we exposed gall bladder cholangiocyte, airway and intestinal organoids to physiological levels of CDCA, to simulate the baseline level of FXR activation that is present in vivo, and infected them with SARS-CoV-2 isolated from a patient’s nasopharyngeal swab^27^ in the absence or presence of UDCA or ZGG (Fig. 1e,f). Suppressing FXR signalling with UDCA or ZGG reduced viral infection in all three types of organoid (Fig. 1e,f and Extended Data Fig. 5a). We then investigated whether the observed reduction in viral infection was a direct result of the FXR-mediated downregulation of ACE2. First, we showed that knockdown of FXR using shRNAs decreases the expression of ACE2 and inhibits viral infection in cholangiocyte organoids independently of the presence of CDCA or that of UDCA or ZGG (Extended Data Fig. 4d). Accordingly, after knockdown, treatment with UDCA or ZGG had no effect on viral infection (Extended Data Fig. 4d). Next, to determine whether the modulation of ACE2 is the only mechanism by which UDCA and ZGG reduce SARS-CoV-2 infection, we treated HEK293T cells that had been genetically engineered to overexpress ACE2 independent of FXR^28^ (Extended Data Fig. 6a,b) with UDCA or ZGG, and then infected them with SARS-CoV-2. As expected, in the absence of ACE2 modulation, UDCA and ZGG did not affect viral replication (Extended Data Fig. 6c). Together, these results confirm that UDCA and ZGG reduce susceptibility to SARS-CoV-2 infection in multiple cell types in vitro through the FXR-mediated regulation of ACE2.

## FXR regulates viral infection in vivo

**Fig. 2:**
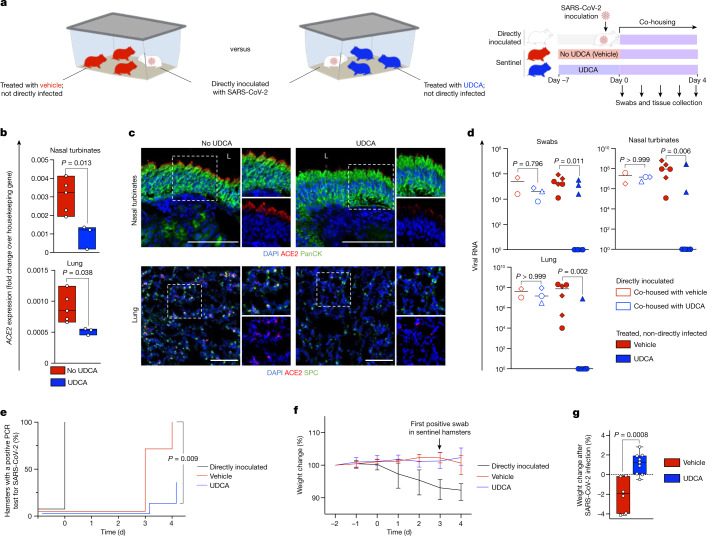
Inhibition of FXR reduces ACE2 expression and SARS-CoV-2 infection in vivo. **a**, Schematic of the experiment performed in Syrian golden hamsters. Sentinel hamsters were not directly inoculated with virus. SARS-CoV-2 infection in sentinel hamsters was achieved through transmission from directly inoculated hamsters after co-housing. **b**, qPCR showing that treatment with UDCA reduces the levels of ACE2 in hamster nasal turbinates and lungs. Housekeeping gene, *Gapdh*; *n* = 5 vehicle (no UDCA) group versus *n* = 3 UDCA group; unpaired two-tailed *t*-test; centre line, median; box, interquartile range; whiskers, range; bars, s.d. **c**, Immunofluorescence images showing the levels of ACE2 in nasal and respiratory epithelium of hamsters receiving UDCA versus vehicle. *n* = 3 hamsters per group. Scale bars, 100 μm. **d**, qPCR showing the levels of SARS-CoV-2 RNA in swabs, nasal turbinates and lungs of directly inoculated hamsters and sentinel hamsters treated with UDCA or vehicle and co-housed with infected hamsters. Samples were collected after four days of co-housing. SARS-CoV-2 nucleocapsid RNA quantification relative to 18s rRNA. *n* = 3 hamsters per group; *n* = 9 UDCA, *n* = 6 vehicle hamsters; hamsters from each experiment are represented with different symbols; Kruskal–Wallis test adjusted for multiple comparisons. **e**, Kaplan–Meier curve showing the percentage of hamsters with a PCR-positive swab for SARS-CoV-2 over the course of the experiment outlined in **a**. *n* = 9 UDCA, *n* = 6 vehicle, *n* = 5 directly inoculated hamsters; log-rank Mantel–Cox test comparing UDCA versus vehicle. **f**, Percentage weight change from the start of the experiment outlined in **a**. Bars, range. Day 0 corresponds to the start of co-housing. **g**, Percentage weight change after SARS-CoV-2 infection in sentinel hamsters. The time of infection was defined as the earliest day on which a sentinel hamster had a positive swab (day 3 for both UDCA and vehicle groups). *n* = 3 independent experiments; *n* = 9 UDCA, *n* = 6 vehicle, *n* = 5 directly inoculated hamsters; unpaired two-tailed *t*-test; centre line, median; box, interquartile range; whiskers, range; bars, s.d. Source data

**Extended Data Fig. 7:**
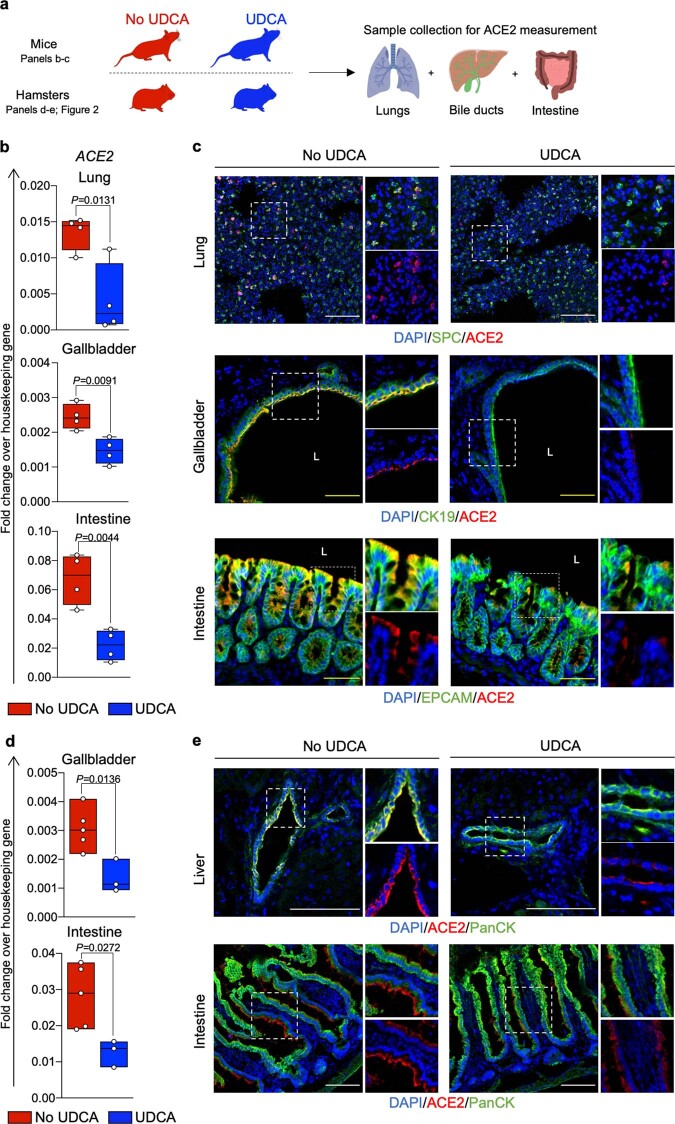
FXR inhibition reduces ACE2 levels in vivo. (**a**) Schematic representation of the experiment performed. (**b**) QPCR showing that treatment with UDCA in FVB/N mice reduces ACE2 levels in lung, gall bladder and intestinal tissue. Housekeeping gene, GAPDH; n = 4 animals per group (UDCA vs. no UDCA control group, see Methods); unpaired two-tailed t-test; centre line, median; box, interquartile range (IQR); whiskers, range; bars, standard deviation. (**c**) Immunofluorescence images showing ACE2 levels upon treatment with UDCA in respiratory, biliary and intestinal epithelium in FVB/N mice. N = 4 mice per group. White scale bars 100 μm; yellow scale bars 50 μm. (**d**) QPCR showing that treatment with UDCA in Syrian golden hamsters reduces ACE2 levels in the gall bladder and intestinal tissue. Housekeeping gene, GAPDH; n = 5 vehicle/No UDCA group vs n = 3 UDCA group; unpaired two-tailed t-test; centre line, median; box, interquartile range (IQR); whiskers, range; bars, standard deviation. (**e**) Immunofluorescence images showing ACE2 levels upon treatment with UDCA in biliary and intestinal epithelium in Syrian golden hamsters. N = 3 hamsters per group. White scale bars 100 μm. Source data

To validate the relevance of these findings in vivo, we assessed the effect of UDCA on ACE2 expression in FVB/N mice and Syrian golden hamsters. We compared the expression of ACE2 in the respiratory, biliary and intestinal epithelia of four mice that were treated with UDCA relative to four control mice that did not receive UDCA (Extended Data Fig. 7a). We repeated the same experiment in Syrian golden hamsters receiving UDCA (*n* = 3 hamsters) and control untreated hamsters (*n* = 5 hamsters) and measured the levels of ACE2 in the nasal, respiratory, biliary and intestinal epithelium of these animals (Fig. 2a). Our results show that treatment with UDCA reduces the expression of ACE2 in mice (Extended Data Fig. 7a–c) and hamsters (Fig. 2a–c and Extended Data Fig. 7d,e).

**Extended Data Fig. 8:**
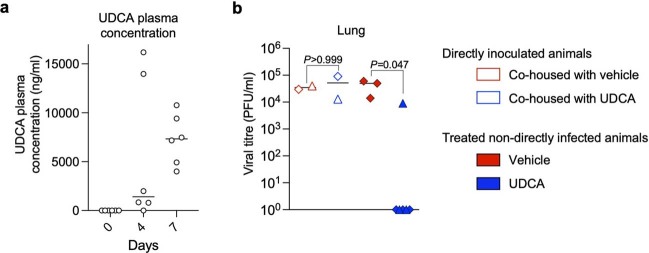
UDCA plasma concentration in vivo. (**a**) UDCA concentration in the plasma of hamsters over 7 days of treatment with 416 mg/kg/day of UDCA. N = 6 animals/group; line, median. (**b**) Viral titre showing levels of infectious virus as measured by plaque assay in lungs in directly inoculated hamsters and sentinel animals treated with UDCA or vehicle and co-housed with infected animals. Samples were collected after 4 days of co-housing. N = 6 UDCA vs n = 3 vehicle animals; Kruskal–Wallis test with Dunn’s correction for multiple comparisons. Animals from each experiment are represented with different symbols; line, median. Source data

To examine whether the UDCA-mediated downregulation of ACE2 reduces SARS-CoV-2 infection in vivo, we used the well-established Syrian golden hamster model of infection. Nine hamsters were treated with UDCA for seven days (UDCA group), to achieve plasma concentrations of UDCA comparable to those in the blood of patients^29^ (Extended Data Fig. 8a). Another six hamsters that received only the vehicle were used as controls (control group). Neither group was directly infected with the virus (sentinel animals). On day 7 we inoculated *n* = 5 independent, healthy hamsters with the SARS-CoV-2 Delta variant (B.1.617.2) via the intranasal route (directly infected animals). Subsequently, each infected hamster was co-housed with a group of *n* = 3 randomly selected sentinel (uninfected) hamsters from the UDCA or the control group for a period of four days to assess SARS-CoV-2 transmission (Fig. 2a). Viral infection in sentinel hamsters was assessed with plaque assays from the lungs collected at the end of the experiment (Extended Data Fig. 8b) and confirmed with daily swabs, and viral qPCR in tissue collected from the lungs and nasal turbinates of the hamsters at the end of the experiment. Our data show that treatment with UDCA prevented the transmission of SARS-CoV-2 in *n* = 6 out of 9 sentinel hamsters (33% infected versus 67% uninfected), whereas SARS-CoV-2 was transmitted in *n* = 6 out of 6 (100%) sentinel hamsters receiving vehicle (*P* = 0.027, Fisher’s exact test) (Fig. 2d,e) for the duration of the experiment. Both directly inoculated and control hamsters lost weight after viral infection, in contrast to UDCA-treated hamsters, which gained weight (Fig. 2f,g), suggesting a milder course of clinical disease in UDCA-treated hamsters. In summary, our in vivo results confirm the chemoprophylactic potential of UDCA against COVID-19.

## FXR regulates infection in human organs

**Fig. 3:**
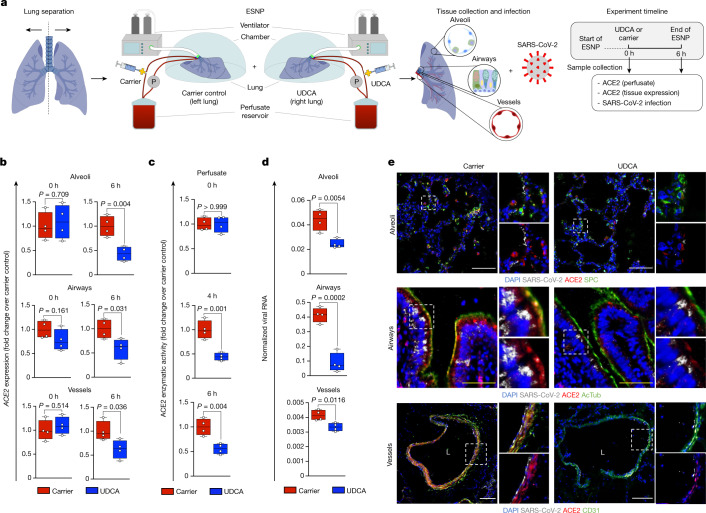
FXR inhibition reduces ACE2 levels and SARS-CoV-2 infection in a human lung ex vivo. **a**, Schematic representation of the lung ESNP experiment, including type of samples and timeline. 0 h: baseline sample collection and UDCA or carrier administration. The 0-h samples were collected before administration of UDCA. 6 h: 6 h after UDCA or carrier administration. For each time point, four independent tissue samples were obtained from the lung parenchyma (alveoli), the airways and the vessels for each lung and used for ACE2 measurement and viral infection (*n* = 4 lung parenchyma, *n* = 4 airway and *n* = 4 pulmonary vessel samples per lung per time point). **b**, qPCR showing that treatment with UDCA reduces the levels of ACE2 in human alveoli, airway and pulmonary vessels perfused ex situ. Housekeeping gene, *GAPDH*; *n* = 4 independent samples; unpaired two-tailed *t*-test; centre line, median; box, interquartile range (IQR); whiskers, range; error bars, s.d. **c**, ACE2 enzymatic activity in the perfusate, showing that UDCA reduces ACE2. *n* = 4 independent samples; unpaired two-tailed *t*-test; centre line, median; box, interquartile range (IQR); whiskers, range; bars, s.d. **d**, qPCR showing that 6 h of ESNP with UDCA reduces SARS-CoV-2 infection in human alveoli, airway and pulmonary vessels ex vivo. Housekeeping gene, *GAPDH*. *n* = 4 independent samples; unpaired two-tailed *t*-test. **e**, Immunofluorescence staining for ACE2 and SARS-CoV-2 in human alveoli, airway and pulmonary vessels after ESNP (6 h) with UDCA or carrier. *n* = 4 independent samples. White scale bars, 100 μm, yellow scale bars, 50 μm. UDCA concentration, 2,000 ng ml^−1^. AcTub, acetylated α-tubulin; L, lumen.

We then looked to validate these observations in whole human organs. We focused initially on the lung as one of the primary sites of SARS-CoV-2 infection. To conduct our experiments, we used a pair of human lungs that was declined for transplantation and performed ex situ normothermic perfusion (ESNP) with clinically appropriate mechanical ventilation to oxygenate the lungs ex vivo (Fig. 3a). ESNP was developed to objectively assess and potentially improve donor organ function, enhance organ preservation and reduce reperfusion injury by perfusing grafts with warm oxygenated blood (packed red cells) or substitute perfusion solution before transplantation^30,31^. This setting ensured that the lungs remained in near-physiological conditions during the experiment^32^. To assess the effect of UDCA, we surgically divided the right and the left lungs from the same donor and connected them to two separate but identical ESNP circuits. This setting allowed us to administer UDCA to one lung (UDCA lung) and use the other lung as a matched control treated with carrier without UDCA (control lung) to facilitate comparison (Fig. 3a).

Immediately before the administration of UDCA, we measured baseline ACE2 expression in both lungs (0-h samples collected from lung parenchyma, airway and pulmonary vessels; *n* = 4 independent samples from each part of the organ per lung, 24 samples in total; Fig. 3b) and ACE2 activity in the circulating perfusate from each ESNP circuit (*n* = 4 independent measurements per circuit; Fig. 3c). We then administered UDCA ‘systemically’ in the perfusate of the UDCA lung; UDCA was diluted in saline to 2,000 ng ml^−1^, corresponding to the steady-state plasma concentration achieved in patients after multiple doses of oral UDCA^29^. At the same time, the control lung received an equal volume of saline (carrier) (Fig. 3a; 0 h refers to the administration of UDCA or carrier). We continued ex situ perfusion with UDCA for 6 h. Repeat perfusate and tissue samples were collected at 6 h, matching our pre-UDCA measurements (*n* = 24 independent tissue samples and *n* = 8 independent perfusate samples per time point). We observed that ex vivo treatment with UDCA reduced the expression of ACE2 in lung parenchyma, airway and pulmonary vessels, and the activity of ACE2 in the perfusate, compared with the carrier control (Fig. 3b,c).

We then assessed the importance of this reduction in ACE2 for susceptibility to SARS-CoV-2 infection. We infected samples from the lung parenchyma, airway and vessels of each lung 6 h after treatment with UDCA or carrier (*n* = 4 parenchymal, *n* = 4 bronchial and *n* = 4 pulmonary vessel independent samples per lung; see Methods) and observed that treatment with UDCA reduced SARS-CoV-2 infection (Fig. 3d,e). These results show that clinical doses of circulating UDCA can downregulate the levels of ACE2 and reduce SARS-CoV-2 infection in human lungs ex vivo.

**Extended Data Fig. 9:**
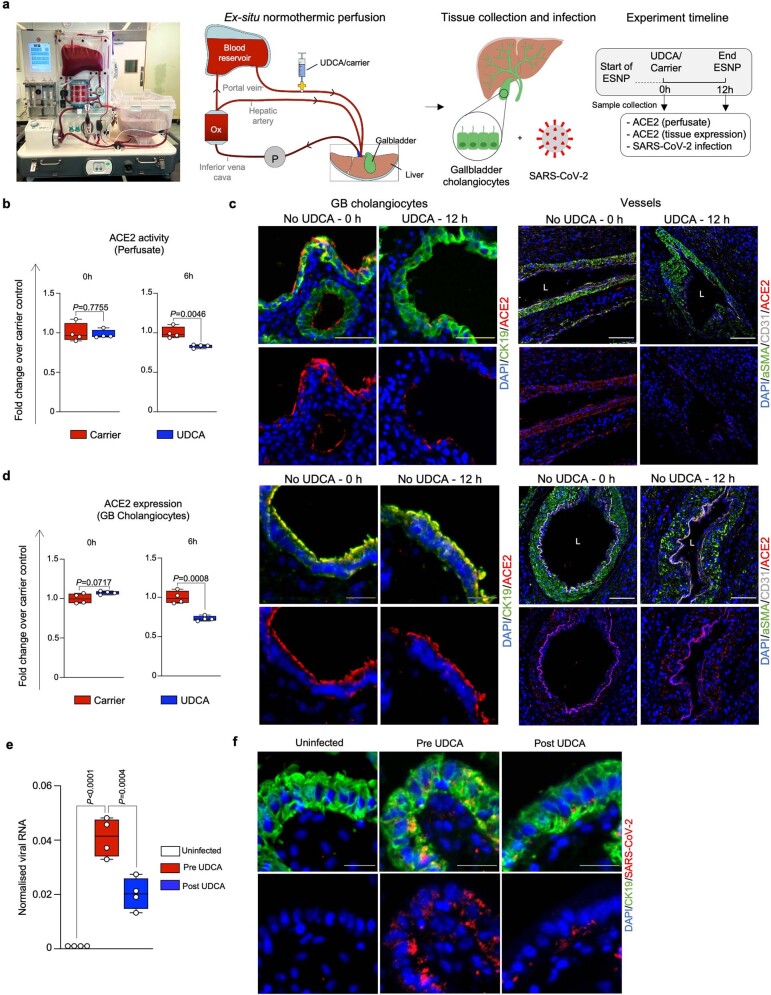
FXR inhibition reduces SARS-CoV-2 infection in human organs ex vivo. (**a**) Photograph and schematic of the liver ex situ normothermic perfusion (ESNP) experiment performed; including type of samples and timeline. 0 h: 0 h baseline sample collection and UDCA/carrier administration. The 0 h samples were collected immediately prior to UDCA administration. 12 h: 12 h after UDCA/carrier administration. For each time point, 4 independent tissue samples were obtained from the grafts’ gall bladder and used for ACE2 measurement and viral infection (n = 4 gall bladder tissue samples per time point). (**b**) ACE2 enzymatic activity measurement showing that ESNP with UDCA reduces ACE2 activity in the circulating perfusate, compared to carrier only control. N = 4 independent samples; unpaired two-tailed t-test; centre line, median; box, interquartile range (IQR); whiskers, range; bars, standard deviation. (**c**) Immunofluorescence images showing ACE2 expression in gall bladder cholangiocytes and cells of the vasculature (smooth muscle and endothelial cells) in human livers before and after ESNP with UDCA/carrier. N = 4 independent samples. White scale bars 100 μm; yellow scale bars 50 μm, grey scale bars 25 μm. (**d**) QPCR demonstrating that ESNP with UDCA reduces ACE2 levels in gall bladder cholangiocytes, compared to carrier only control. Housekeeping gene, HMBS; n = 4; unpaired two-tailed t-test; centre line, median; box, interquartile range (IQR); whiskers, range; bars, standard deviation. (**e-f**) QPCR (**e**) and immunofluorescence (**f**) showing that UDCA reduces SARS-CoV-2 infection in human gall bladder ex vivo. Housekeeping gene, GAPDH; n = 4; one-way ANOVA adjusted for multiple comparisons; centre line, median; box, interquartile range (IQR); whiskers, range; bars, standard deviation. Scale bars 25 μm. (UDCA, concentration, 2,000 ng/ml).

FXR inhibitors are metabolized by the liver, and ultimately distributed to different tissues through the systemic circulation. To simulate this process, we repeated ESNP with two human liver grafts (Extended Data Fig. 9a; see Methods). One liver was perfused with UDCA (2,000 ng ml^−1^); the other was perfused with carrier and served as a control. In keeping with our lung findings, we observed that ‘systemic’ treatment with UDCA decreased the levels of ACE2 in the circulating perfusate (Extended Data Fig. 9b) and in gall bladder cholangiocytes (*n* = 4 independent samples from the grafts’ gall bladder per time point) (Extended Data Fig. 9c,d) and reduced SARS-CoV-2 infection in gall bladder cholangiocytes (Extended Data Fig. 9e,f). These results confirm that suppression of FXR signalling through systemic administration of the approved drug UDCA can downregulate ACE2 in the circulating perfusate and tissue (lung parenchyma, bronchi, vessels and gall bladder epithelium) of machine-perfused organs and reduce SARS-CoV-2 infection ex vivo.

## UDCA reduces ACE2 in humans

**Fig. 4:**
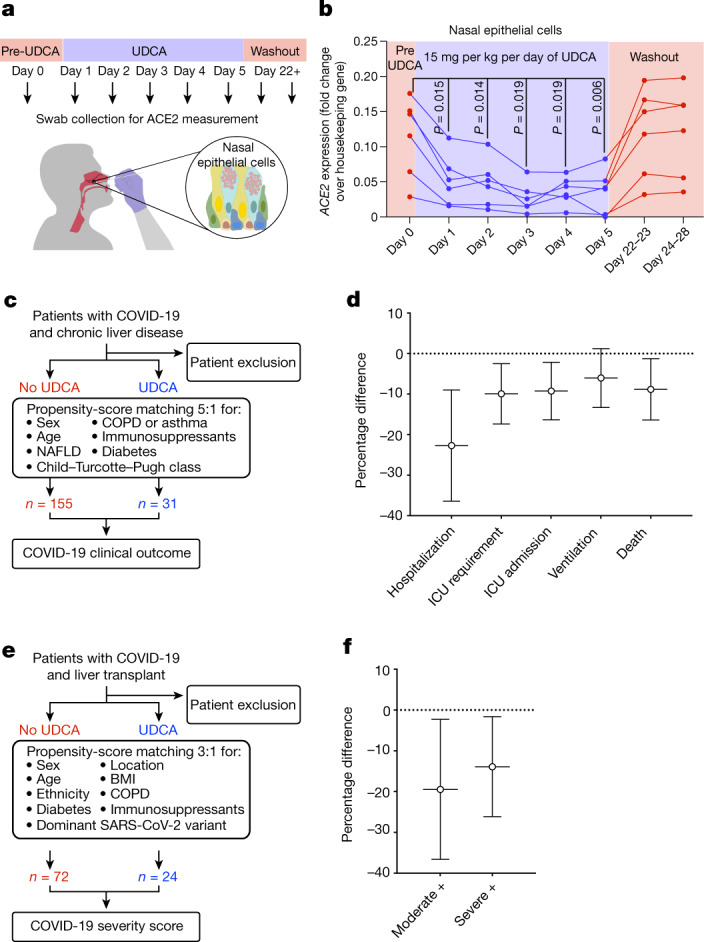
UDCA is associated with lower levels of ACE2 and a better clinical outcome in patients with COVID-19. **a**,**b**, Schematic representation of the study design. Six healthy individuals received 15 mg per kg per day of UDCA for 5 days. ACE2 levels were measured by qPCR in nasal epithelial cells collected with nasopharyngeal swabs. Day 0 corresponds to samples collected immediately before starting UDCA treatment. Samples were collected daily during drug administration and again at day 22–23 and 24–28 to assess the washout of UDCA. **b**, qPCR measurement of the levels of *ACE2* in nasal epithelial cells collected with nasopharyngeal swabs. Each dot represents one individual measurement; lines connect dots from the same individual (*n* = 6). Housekeeping gene, *GAPDH*; *n* = 6 individuals; one-way ANOVA with Geisser–Greenhouse correction. See Supplementary Table 5 for participant characteristics. **c**, Schematic overview of the analysis performed in the exploratory cohort corresponding to **d** (see Methods). **d**, Propensity-score-matched analyses showing major outcomes after SARS-CoV-2 infection in patients taking UDCA compared to control individuals not taking UDCA. *n* = 155 patients not on UDCA; *n* = 31 patients on UDCA. See Supplementary Tables 6 and 7 for patient characteristics. Bars, 95% CI. ICU, intensive care unit. **e**, Schematic overview of the analysis performed in the validation cohort corresponding to **f** (see Methods). **f**, Propensity-score-matched analyses showing disease severity after SARS-CoV-2 infection in patients taking UDCA compared to control individuals not taking UDCA, using the NIH COVID-19 severity score. Moderate +, moderate, severe or critical disease; severe +, severe or critical disease. *n* = 72 patients not on UDCA; *n* = 24 patients on UDCA. See Supplementary Table 8 for patient characteristics. Bars, 95% CI.

Our previous results encouraged us to assess the potential effect of UDCA on the levels of ACE2 in humans. Given the favourable safety profile, lack of side effects and limited cost of UDCA, we recruited eight volunteers from the University Medical Centre Hamburg-Eppendorf and treated them with UDCA at the standard therapeutic dosage of 15 mg per kg per day^26^ for 5 days (Supplementary Table 5). The nasal epithelial cells of the volunteers were collected using nasopharyngeal swabs, and the levels of ACE2 were measured at multiple time points before, during and after treatment with UDCA (see Methods and Fig. 4a). Participants with non-detectable cellular RNA in their nasopharyngeal swabs were excluded (*n* = 2). Our results showed that in humans, UDCA reduces the levels of ACE2 in the nasal epithelium, which is a prime site of SARS-CoV-2 infection (Fig. 4b).

**Extended Data Fig. 10:**
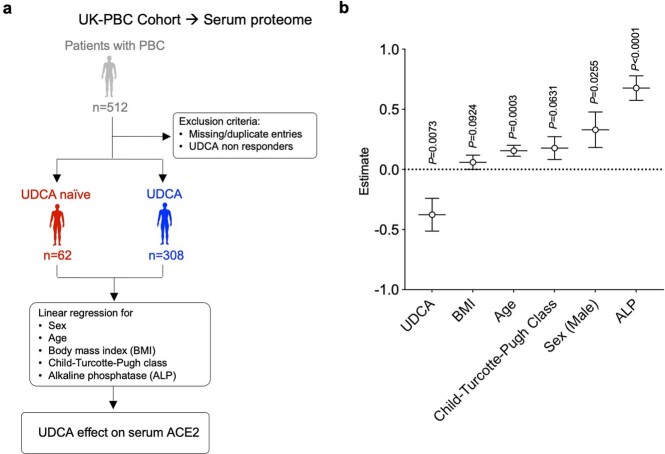
UDCA is associated with lower levels of ACE2 in patients with PBC. (**a**) Schematic illustrating the patient cohorts compared in (b). (**b**) Multiple linear regression analysis of serum ACE2 demonstrates that UDCA correlates with lower ACE2 levels in patients with primary biliary cholangitis (PBC) receiving UDCA (n = 308) vs. patients with PBC who were naïve to treatment (n = 62). Values plotted are β coefficients. (BMI, body mass index; ALP, alkaline phosphatase). Bars, 95% CI.

To further confirm our findings, we took advantage of the fact that UDCA is extensively used in patients with cholestatic liver disorders (for example, as a first-line treatment for the cholestatic autoimmune disorder PBC). We interrogated a published serum proteomics dataset from the UK-PBC patient cohort^33^, comparing the levels of ACE2 in the serum of patients who had not been treated with UDCA (*n* = 62) and patients who had received UDCA (*n* = 308). We observed that UDCA correlates with lower serum levels of ACE2 after linear regression for age, sex, body mass index (BMI), stage of liver disease (Child–Turcotte–Pugh class) and alkaline phosphatase (ALP) (*P* = 0.007; Extended Data Fig. 10a,b; see Methods), validating our previous findings (Supplementary Table 5 and Supplementary Information).

## UDCA may improve COVID-19 outcome

On the basis of these observations, we decided to investigate the possible effect of UDCA treatment on the outcome of COVID-19 in patients. For this, we analysed the COVID-Hep and SECURE-Liver registries^34,35^. These registries comprise data from patients with chronic liver disease (*n* = 1,096) who developed COVID-19, including patients with cholestatic liver disorders who had been treated with UDCA (*n* = 31) (Fig. 4c and Supplementary Tables 7 and 8). We observed that—accepting the potential for selection bias in case reporting—patients who were treated with UDCA had better outcomes compared to patients who did not receive UDCA, including reduced hospitalization, admission to intensive care units and death (Fig. 4d), after propensity-score matching (no UDCA:UDCA = 5:1) for sex, age, diabetes, stage of liver disease (Child–Turcotte–Pugh class), immunosuppression, chronic pulmonary disease and non-alcoholic fatty liver disease (Fig. 4d). We note that propensity-score matching was not possible for alcohol-related liver disease (ARLD), so these patients were excluded from the analysis (see Methods). We then sought to replicate these results in a second independent patient cohort. For this, we interrogated liver-transplant recipients in the Veterans Outcomes and Costs Associated with Liver disease (VOCAL) cohort who received at least two doses of a COVID-19 mRNA vaccine. Of 119 vaccinated participants who developed COVID-19, 24 were receiving UDCA (Supplementary Table 9). These 24 participants on UDCA were matched with 72 who were not on UDCA (no UDCA:UDCA = 3:1) for sex, age, ethnicity, BMI, location within the United States, diabetes, chronic pulmonary disease, the type of immunosuppression (calcineurin inhibitor therapy, with or without anti-metabolite therapy) and the dominant SARS-CoV-2 variant at the time of infection (Fig. 4e). We found that, again accepting the potential for selection bias in case reporting, patients on UDCA were less likely to develop moderate, severe or critical COVID-19 (*P* = 0.026; Fig. 4f) according to the National Institutes of Health (NIH) COVID-19 severity score^36^. During the publication of this manuscript, we became aware of an independent study investigating the association between exposure to UDCA and outcomes of COVID-19 among participants in the VOCAL cohort with cirrhosis^37^. In this analysis, 1,607 participants with cirrhosis and UDCA exposure were propensity-score-matched with 1,607 participants with cirrhosis but without UDCA exposure. The authors found, using multivariable logistic regression, that UDCA exposure was associated with 46% reduced odds of developing COVID-19 (adjusted odds ratio (aOR) 0.54, 95% confidence interval (CI) 0.41–0.71, *P* < 0.0001). The association was observed across the spectrum of COVID-19 against symptomatic illness (aOR 0.54, 95% CI 0.39–0.73, *P* < 0.0001), at least moderate COVID-19 (aOR 0.51, 95% CI 0.32–0.81, *P* = 0.005) and severe or critical COVID-19 (aOR 0.48, 95% CI 0.25–0.94, *P* = 0.03). These results provide additional independent evidence that reinforces the results of our study. Together, the findings from our exploratory and validation cohorts suggest that the effects of UDCA on the clinical outcomes of COVID-19 should be further investigated in a large prospective clinical trial.

## Discussion

Considered collectively, our results show that FXR participates in the regulation of ACE2 expression in several tissues that are involved in SARS-CoV-2 replication. Suppressing FXR activity, using the clinically approved drug UDCA, downregulates the expression of ACE2 and reduces SARS-CoV-2 infection in vitro, in vivo and ex vivo. Furthermore, our clinical observations indicate that UDCA reduces the levels of ACE2 in the nasal epithelium of healthy individuals and suggest a correlation between UDCA and positive clinical outcomes in patients with COVID-19.

The finding that FXR regulates ACE2 is not entirely surprising. The functions of ACE2 as a molecular chaperone for the amino acid transporter SLC6A19 and as a peptidase justify its presence in the gastrointestinal tract and suggest that it has a role in digestion. Accordingly, the upregulation of ACE2 expression by FXR—which is activated by bile, a digestive fluid—may reflect a mechanism to increase peptidase levels and amino acid absorption during digestion. Moreover, in addition to its role in the gastrointestinal system, FXR is expressed in multiple organs, including the lungs^20,21^, with a broad variety of functions, ranging from bile acid^22^ and lipid metabolism^38^ to glucose homeostasis^39^, fibrosis^40^ and inflammation^41^. Notably, its natural ligands, such as bile acids and hormones^42^ (for example, androgens), are present in the systemic circulation^22^ and it is the therapeutic target of several approved drugs^17^. This broad expression and function explains how FXR could regulate ACE2 in tissues beyond those of the biliary tree.

Our results reveal the potential of ACE2 modulation as a host-directed treatment that might be efficacious as primary and secondary prophylaxis in COVID-19. These findings are in keeping with existing studies that show the benefits of targeting the virus–host interaction for SARS-CoV-2 at the level of ACE2 (ref. ^1^) or the spike protein^7^. Indeed, large Mendelian randomization analyses in more than 7,554 patients admitted to hospital with COVID-19 and more than one million control individuals found that higher levels of ACE2 were strongly correlated with an increased risk of COVID-19-related hospitalization, identifying ACE2 as a logical candidate for drug development in COVID-19 (ref. ^1^). In addition, the extensive clinical literature that supports Ronapreve and Evusheld—both dual combinations of monoclonal antibodies against the SARS-CoV-2 spike protein—shows the utility of inhibiting the viral spike protein–ACE2 interaction for prophylaxis and treatment in COVID-19 for susceptible pre-Omicron variants^43,44^. However, targeting the viral spike protein with monoclonal antibodies is limited by the diversity and evolution of the viral spike sequences, which renders many of these agents ineffective against new SARS-CoV-2 variants^7^. By contrast, targeting ACE2 is advantageous for several reasons. ACE2 modulation is a host-directed treatment, which does not target the virus. Such mechanisms may present a higher barrier to the emergence of resistance, although this has yet to be empirically demonstrated. Furthermore, because ACE2 is a critical mechanism for cell entry, the approach may be more resilient as variants continue to emerge^45^. Finally, ACE2 is a common receptor for multiple coronaviruses, such as SARS-CoV and HCoV-NL63. Confirmation of the efficacy of this strategy may therefore provide a quickly deployable intervention in the event of future coronavirus outbreaks. Taken collectively with our own observations on the effects of ACE2 modulation for SARS-CoV-2 infection, these points indicate that ACE2 modulators warrant consideration as priority candidates for clinical evaluation in COVID-19 trials^1^.

Our finding that the suppression of FXR signalling through UDCA or ZGG reduces ACE2 expression and limits SARS-CoV-2 infection identifies a potential clinical application for FXR inhibitors, but also raises some points for consideration. First, FXR activation decreases inflammation by modulating NF-κB in several organs^46^, including the lungs^21^, liver^17^ and intestine^17^. Conversely, UDCA has been shown to reduce inflammation in multiple tissues, including the lung, in an FXR-independent manner^27^. Given the complex interplay among FXR, UDCA and inflammation^47^, the balance of benefits for FXR activation in terms of SARS-CoV-2 infection and inflammation should be carefully considered. It is possible that FXR suppressors beyond UDCA, which lack anti-inflammatory effects, would be better suited for prophylaxis or early intervention and not indicated for severe disease with ongoing tissue inflammation^48^. Second, our study suggests that FXR activators that are used in clinical practice, such as obeticholic acid, may increase the risk of developing COVID-19 by upregulating ACE2 in healthy individuals. Paradoxically, in patients with liver disorders, obeticholic acid may prevent COVID-19 by reducing disease severity and ameliorating cholestasis, resulting in a net reduction in FXR activity. Therefore, further studies are needed to clarify these points.

Our results identify UDCA as a particularly advantageous modulator of the levels of ACE2 for use in COVID-19. We have shown that UDCA reduces SARS-CoV-2 infection in vitro, in vivo and ex vivo, and that it reduces the expression of ACE2 in the nasal epithelium of healthy volunteers. Although our animal data do not exclude the possibility that UDCA delays the transmission of SARS-CoV-2 beyond the duration of our experiments, our patient data show that this does not change the net effect of reducing disease severity, thus making UDCA a particularly attractive candidate for investigation as pharmacological prophylaxis against SARS-CoV-2 infection. Compared to other agents, such as vaccines and monoclonals, UDCA is easy to administer orally, easily stored, affordable and accessible to health systems worldwide for large-scale production, as it is off patent. In addition, UDCA is well-tolerated and has limited drug–drug interactions and a favourable safety profile, enabling it to be administered for long periods of time. Of note, UDCA is already administered over long periods for different clinical indications to vulnerable groups who would benefit from chemoprophylaxis, such as individuals who have received bone marrow or liver transplants (for the prevention of veno-occlusive disease^49^ and for the treatment of cholangiopathy^26^, respectively). It has excellent tolerability and minimal side effects in these groups of patients^26,49^, which shows the feasibility of using UDCA as pharmacological prophylaxis against COVID-19 in vulnerable groups. Nevertheless, our study is not a clinical trial and therefore we cannot exclude the potential for confounding and selection biases. Consequently, it will be imperative to validate these results in prospective double-blinded clinical trials and to fully assess the effect of this drug on ACE2 levels and susceptibility to SARS-CoV-2 infection. For the avoidance of doubt, the authors do not support the use of UDCA for COVID-19 until appropriate policy informed by robust clinical evidence is available. The authors also do not condone the use of UDCA as a substitute for highly effective vaccinations in patients for whom they are indicated.

Finally, we have shown that UDCA an reduce ACE2 levels and SARS-CoV-2 infection in machine-perfused organs. This is one of the first studies to test the effect of a drug in a whole human organ perfused ex situ. This finding could prove important for organ transplantation, especially given concerns about peri-operative viral transmission^50^. Furthermore, although more data are required to definitively establish this approach, our work sets the stage for future studies using machine-perfused organs for pharmacological studies.

In conclusion, these results validate CDCA-treated cholangiocyte organoids as a platform for disease modelling and drug testing against SARS-CoV-2 infection, identify FXR as a therapeutic target in the management of COVID-19 and open up new avenues for the modulation of ACE2 through FXR for the prevention of infection with SARS-CoV-2 as well as other viruses that use ACE2 for cell entry.

## Methods

### Ethical approval

All human samples were obtained from patients, deceased transplant organ donors or liver explants with informed consent for use in research and ethical approval (Research Ethics Committee (REC) 09/H0305/68, 14/NW/1146, 15/EE/0152, 15/WA/0131 and 18/EE/0269; and Papworth Hospital Research Tissue Bank project number T02233). The mouse study was approved by the Animal Ethics Committee of the Medical University of Vienna and the Federal Ministry of Science, Research and Economy (BMWFW-66.009/0008-WF/3b/2015) and was performed according to the Animal Research: Reporting of In Vivo Experiments (ARRIVE) guidelines. The hamster animal study was approved by the University of Liverpool Animal Welfare and Ethical Review Board and performed under UK Home Office licences (PP9284915 and PP4715265) and it was completed at the University of Liverpool and conducted in accordance with the UK Home Office Animals Scientific Procedures Act (ASPA, 1986). Human lungs and livers retrieved for transplantation but subsequently declined were used for ESNP experiments (National Research Ethics Committee (NREC) North East—Newcastle and North Tyneside 1 16/NE/0230, lung; NREC East of England—Cambridge East 14/EE/0137, liver). The study involving volunteers from the University Medical Centre Hamburg-Eppendorf was performed with informed consent and ethical approval (Ethik-Kommission der Ärztekammer Hamburg; ref. no. 2021-300121-WF). The COVID-Hep.net and SECURE-Liver registries data were deemed not to constitute human research by Clinical Trials and Research Governance at the University of Oxford (https://covid-hep.net/img/CTRG_COVID-Hep_20200402.pdf) and by the Institutional Review Board of University of North Carolina (https://covidcirrhosis.web.unc.edu/faq/), respectively. The study involving patients from the VOCAL cohort was performed with informed consent and ethical approval from the Miami VA Institutional Review Board (unique study approval ID 1477437-22).

### 10X single-cell RNA sequencing, data analysis and availability

We used our previously published single-cell RNA sequencing (scRNA-seq) dataset including primary cholangiocytes, cholangiocyte organoids (COs) originating from different regions of the biliary tree (intrahepatic ducts, common bile duct and gall bladder) and the same organoids after bile treatment. Tissue dissociation, cell isolation, 10X single-cell library preparation and 10X data processing, normalization and analysis was performed as previously described^14^. The 10X raw data (fastq files) have been deposited in the repository ArrayExpress with the accession number E-MTAB-8495. scRNA-seq data were analysed using Anaconda-Navigator v.1.9.12, Jupyter Notebook v.6.0.3 and Rstudio v.1.1.463.

### Human tissue collection and processing

Human primary tissue was obtained from biopsies, deceased organ donors or liver explants after obtaining informed consent. Depending on the application, primary fresh tissue was embedded in optimal cutting temperature (OCT) compound and stored at −80 °C; or fixed in 10% formalin, dehydrated and embedded in paraffin. Sections from embedded tissue were cut at a thickness of 5–10 μm using a cryostat or a microtome and mounted on microscopy slides for further analysis.

### Bile sample collection and processing

Human bile was collected during endoscopic retrograde cholangio-pancreatography (ERCP) or intraoperatively with informed consent from the patient. For viral RNA quantification, samples were immediately lysed using an equal volume of RNA lysis buffer (Sigma) and stored at −20 °C.

### Cell culture

Primary cholangiocytes were isolated and COs were derived and cultured using our established methodology^14,16,51^. COs obtained from intrahepatic duct (IHD), common bile duct (CBD) and gall bladder (GB) tissue were used in this study. Cholangiocytes derived from any of the different regions of the biliary tree (IHD, CBD or GB) acquired a common gall bladder identity when treated with CDCA, as previously reported^14^. The experiments described were performed with COs derived from all the three regions of the biliary tree (IHD, CBD and GB) and provided congruent results. For consistency, the results shown correspond only to COs derived from the gall bladder (gall bladder cholangiocyte organoids—GCOs).

Human primary intestinal organoids, derived from terminal ileum biopsies, were provided by S.J.A.B.’s group. The organoids were derived following a modification of previously described protocols^52^, embedded in Matrigel and cultured in Intesticult (StemCell Technologies) supplemented with penicillin–streptomycin and Rho kinase inhibitor (Stratech Scientific).

Human primary airway organoids were provided by J.-H. L.’s laboratory. The organoids were derived and cultured as previously described^24^.

Vero E6 cells (ATCC CRL—1586), HEK293 cells (ATCC CRL—1573) and HEK293T cells (ATCC CRL—3216) were grown on tissue culture plates or T25 flasks in 10% FBS DMEM supplemented with l-glutamine and penicillin–streptomycin as previously described^53^. All cell cultures were routinely tested for mycoplasma.

### Availability of biological materials

Detailed protocols for the derivation of primary organoids have been previously reported^24,51^. Cell lines are available from standard commercial sources (https://www.lgcstandards-atcc.org: Vero E6 cells, ATCC CRL—1586; HEK293 cells, ATCC CRL—1573).

### Modulation of FXR activity

CDCA and UDCA were purchased from Sigma-Aldrich (C9377-5G and U-5127-5G), and ZGG was purchased from Santa Cruz (sc-204414) and reconstituted following the manufacturer’s instructions. To modulate FXR activity, organoids were incubated with a final concentration of 10 μM CDCA, or 10 μM CDCA in combination with 10 μM of UDCA or ZGG.

### FXR knockdown

FXR knockdown was performed in COs using commercially available lentiviral particles carrying shRNA gene silencer sequences against the human FXR (*NR1H4*) transcript (Santa Cruz; sc-38848-V). Commercially available lentiviral particles carrying control (scrambled) shRNA sequences (Santa Cruz; sc-108080) were used as control. Successfully transduced COs were selected with puromycin 24 h after viral transduction. Quantification of FXR, ACE2 and SHP expression and SARS-CoV-2 infection was performed 10 days after FXR knockdown.

### ChIP

Approximately 6 × 10^6^ cells were used for each ChIP, and cells were incubated with fresh medium with 100 μM of CDCA, UDCA or ZGG 2 h before collection. ChIP was performed using the True Micro ChiP kit (Diagenode C01010130) according to the manufacturer’s instructions. In brief, following pre-clearing, the lysate was incubated overnight with the FXR antibody (Santa Cruz sc-25309 X) (Supplementary Table 1) or non-immune IgG. ChIP was completed and immunoprecipitated DNA was purified using MicroChip DiaPure columns (Diagenode C03040001). Samples were analysed by qPCR using the ΔΔ*C*_t_ approach as previously described^51^ (see Supplementary Table 3 for primer sequences). Primers flanking the FXRE on the well-known FXR target gene *OSTα* (also known as *SLC51A*; ref. ^54^) were used as a positive control, whereas primers flanking a site distant from the FXRE on the *ACE2* promoter were used as a negative control. The results were normalized to the enrichment observed with non-immune IgG ChIP controls.

### Luciferase reporter

Two different fragments containing the FXRE IR-1 in the *ACE2* gene and in the *SHP* gene (also known as *NR0B2*) were amplified using human genomic DNA as a template and inserted onto a pGL3-promoter luciferase vector. The ACE2 and SHP IR-1 mutants were generated using a site-directed mutagenesis approach (New England BioLabs E0554S). Sequences of primers used are reported in Supplementary Table 4. These gene reporter constructs were co-transfected with a commercially available FXR expression plasmid (OriGene, SC329876) into HEK293 cells using TransIT-293 Transfection Reagent (MirusBio). Twenty-four hours after transfection, cells were treated with 50 μM of CDCA, UDCA and ZGG in fresh medium for 8 h. Luciferase activity was determined with the GLO-Luciferase Reporter Assay System (Promega, Madison, ONE-Glo Luciferase Assay System) and values were normalized to the empty pGL3 vector.

### Immunofluorescence, RNA extraction and qPCR

Immunofluorescence, RNA extraction and qPCR were performed as previously described^14–16^. A complete list of the primary and secondary antibodies used is provided in Supplementary Table 1. A complete list of the primers used is provided in Supplementary Table 2.

All qPCR data were obtained using a QuantStudio 5 384 Well Block (Thermo Fisher Scientific). All qPCR data are presented as the median, IQR and range (minimum to maximum) of four independent experiments unless otherwise stated. Values are relative to the housekeeping gene hydroxymethylbilane synthase (*HMBS*) or glyceraldehyde-3-phosphate dehydrogenase (*GAPDH)*. Statistical analysis is described in the relevant section.

For comparative immunofluorescence images, the cells or sections being compared were stained simultaneously, using the same primary and secondary antibody master mix. All immunofluorescence images were acquired using a Zeiss LSM 700 or 710 confocal microscope using ZEN 2011 SP7 (Zeiss). The same laser power and exposure settings were used to acquire comparative images. Imagej 2.0.0-rc-69/1.53f software (W. Rasband, http://imagej.nih.gov/ij) was used for image processing. Each immunofluorescence image is representative of at least three different experiments.

### Flow cytometry analyses

Flow cytometry in organoids was performed as previously described^51^. In summary, organoids were collected using Cell Recovery Solution (Corning) for 20 min at 4 °C and were then centrifuged at 444*g* for 4 min and dissociated to single cells using StemPro Accutase (Invitrogen). Cells were subsequently fixed using 4% paraformaldehyde (PFA) for 20 min at 4 °C. All flow cytometric analyses were performed on a BD LSR-II flow cytometer (BD Biosciences) using BD FACS Diva 8.0.3 (BD Bioscience) and analysed using FlowJo v.10.4.2. The gating strategy is provided in Supplementary Fig. 1.

### Dose–response curves for ACE2

Primary organoids were treated with 0.01 μM–1 mM of CDCA, UDCA or ZGG and ACE2 expression was measured by qPCR. The inhibitory effect of UDCA and ZGG on FXR activation was assessed on cells treated with 10 μM of CDCA. Data were analysed using the Sigmoidal, 4PL, X is log(concentration) function in GraphPad Prism.

### Cytotoxicity and viability

Primary organoids were treated with 0.1 μM–100 μM of CDCA, UDCA or ZGG and the percentage of viable cells was counted using trypan blue and a Countess II cell counter (Thermo Fisher Scientific). Cellular viability in primary organoids treated with 10 μM of CDCA, UDCA or ZGG was measured using the resazurin-based assay PrestoBlue (Invitrogen, A13261) using SoftMax Pro 5.4.4 on a SpectraMax M2 (Molecular Devices).

### SARS-CoV-2 isolate

The SARS-CoV-2 virus used in this study is the clinical isolate named SARS-CoV- 2/human/Liverpool/REMRQ0001/2020 (ref. ^53^) derived from a patient’s nasopharyngeal swab and isolated by L. Turtle, D. Matthews and A. Davidson, and the Delta lineage (B.1.617.2) hCoV-19/England/SHEF-10E8F3B/2021 (GISAID accession number EPI_ISL_1731019) was provided by W. Barclay through the Genotype-to-Phenotype National Virology Consortium (G2P-UK).

### SARS-CoV-2 infection

All work with infectious SARS-CoV-2 was performed under containment level 3 (CL-3) conditions either at the Cambridge institute of Therapeutic Immunology and Infectious Diseases (CITIID) or at the Centre for Excellence and Long-acting Therapeutics (CELT). SARS-CoV-2 was gifted to the users of the CITIID CL-3 by I. Goodfellow^55,56^ and propagated on Vero E6 cells as previously described^53^. Viral titration was determined using the TCID50 method on Vero E6 cells^53^. For viral infection primary organoids were passaged and incubated with SARS-CoV-2 in suspension at a multiplicity of infection (MOI) of 1 for 2 h. Subsequently, the infected organoids were washed twice with 10 ml of culture medium to remove the viral particles. Washed organoids were plated in 40-μl Matrigel domes, cultured in organoid medium and collected at different time points.

To test whether SARS-CoV-2 produced by infected COs retained its infective capacity, the supernatant from infected COs was collected 24 h after infection and used to infect a fresh batch of SARS-CoV-2 naive organoids.

### Fixation of SARS-CoV-2 infected organoids or tissue

Organoids for immunofluorescence were cultured on coverslips and placed at the bottom of the wells of a 24-well plate. The culture medium was aspirated and replaced with 500 µl of 8% PFA for a minimum of 30 min. After fixation, the coverslips were recovered and transferred to a clean plate, and fresh PBS was added. Primary tissue was fixed for a minimum of 4 h with 8% PFA and then transferred to a clean plate with fresh PBS.

### Quantification of viral infection

Organoids or primary tissue were infected in 24-well plates as described above. Total RNA samples were prepared by adding 500 µl of lysis buffer (25 mM Tris-HCl + 4 M guanidine thiocyanate with 0.5% β-mercaptoethanol) to each well and transferring the lysate (1 ml) to a 5-ml Eppendorf tube. Tubes were vortexed, and 100% analytical grade ethanol was added to a final concentration of 50%. After 10 min of incubation, 860 µl of lysis buffer (containing MS2 bacteriophage as an internal extraction and amplification control) was added and thoroughly mixed. The RNA was then isolated using an RNA spin column as previously described^57^. Viral replication was quantified using qPCR for the expression of the viral RNA-dependent RNA polymerase (*RdRp*) gene with primers specific for a 222-bp fragment from a conserved region of the gene. *GAPDH* was used as a housekeeping gene and MS2 was used as an internal reference as previously described^57^. Viral load was determined relative to *GAPDH*. The sequences of primers and probes used are provided in Supplementary Table 2.

### Transmission electron microscopy

Infected organoids were fixed in 4% paraformaldehyde; 2.5% glutaraldehyde in 0.1 M sodium cacodylate buffer overnight at 4 °C, washed and stored in 0.1 M sodium cacodylate buffer before processing. Samples were post-fixed in 1% aqueous osmium tetroxide (TAAB) and 1.5% potassium ferricyanide overnight at 4 °C, washed thoroughly in dH_2_O and en-bloc-stained in 3% aqueous uranyl acetate (Agar Scientific) for 24 h at 4 °C. Samples were dehydrated through an ethanol series, infiltrated with 1:1 propylene oxide:resin (TAAB) and blocks of fresh resin polymerized at 60 °C for 48 h. Ultrathin sections of around 60 nm were cut from blocks using an EM UC7 ultramicrotome (Leica Microsystems) and mounted on copper grids coated with carbon and formvar (Agar Scientific). Grids were post-stained in uranyl acetate and lead citrate, imaged using a HT7800 transmission electron microscope (Hitachi High Technologies) operating at 100 kV and acquired using HT7800 TEM operating software v.01.21 (Hitachi).

### HEK293 cells stably expressing ACE2

HEK293T cells stably expressing ACE2 were generated as previously described^28^. In brief, HEK293T cells transduced with ACE2 under the control of the spleen focus-forming virus (SFFV) promoter were sorted for high cell-surface ACE2 expression and single-cell-cloned. After expansion, a clone with stable, homogeneously high expression of ACE2 was selected by fluorescence-activated cell sorting (FACS).

### Luciferase reporter for SARS-CoV-2 replication

A luciferase reporter for SARS-CoV-2 protease activity during viral replication was generated as previously described^28^ In brief, HEK293T reporter cells stably expressing ACE2, renilla luciferase (Rluc) and SARS-CoV-2 papain-like protease-activatable circularly permuted firefly luciferase (FFluc) were seeded in flat-bottomed 96-well plates. The following morning, cells were treated with the indicated doses of CDCA, UDCA and ZGG, and infected with SARS-CoV-2 at a MOI of 0.01. The SARS-CoV-2 RdRp inhibitor remdesivir and a neutralizing antibody cocktail blocking the interaction between SARS-CoV-2 spike and ACE2 (REGN-COV2) were included as positive controls. After 24 h, cells were lysed in Dual-Glo Luciferase Buffer (Promega, E2920) diluted 1:1 with PBS and 1% NP-40. Lysates were then transferred to opaque 96-well plates, and viral replication quantified as the ratio of FFluc/Rluc activity measured using the Dual-Glo kit (Promega) according to the manufacturer’s instructions. FFluc/Rluc ratios were expressed as a fraction of the maximum, then analysed using the Sigmoidal, 4PL, X is log(concentration) function in GraphPad Prism.

### Animal studies

#### Mice

The experiments were performed in accordance with the Animal Research: Reporting of In Vivo Experiments (ARRIVE) guidelines and approved by the Animal Ethics Committee of the Medical University of Vienna and the Federal Ministry of Science, Research and Economy (BMWFW-66.009/0008-WF/3b/2015). Friend Virus B NIH (FVB/N) mice were bred in house. Mice were housed in a 12 h–12 h dark–light cycle, with a humidity of 45–65% and temperature of 20–24 °C. Chow was obtained from SAFE—Scientific Animal Food & Engineering (product number A04). Age-matched female mice were used. Mice were assigned randomly to treatment and control groups. Mice in the treatment group received chow supplemented with 1% w/w UDCA and 1% w/w cholic acid, whereas mice in the control group received chow supplemented with 1% w/w cholic acid^58^. Cholic acid was used to activate FXR and study the effects of UDCA on FXR activation^19^. The mice were fed ad libitum for seven days. Data were analysed blinded to the identity of the experimental groups.

#### Hamsters

The experiments were performed in accordance with the UK Home Office Animals Scientific Procedures Act (ASPA, 1986). In addition, all studies were approved by the University of Liverpool Animal Welfare and Ethical Review Board and performed under UK Home Office licences PP9284915 and PP4715265. Golden Syrian hamsters were purchased from Janvier Labs. Hamsters were housed in a 12 h–12 h dark–light cycle, with a humidity of 45–65% and temperature of 20–24 °C. Age-matched male hamsters were used, weighing between 80 g and 100 g. Hamsters were assigned randomly to treatment and control groups. Hamsters in the treatment groups received a daily oral regimen of UDCA (416 mg per kg) by oral gavage, whereas those in the control group received vehicle only. The hamsters were fed ad libitum and treatment continued for seven days to achieve a similar blood concentration of UDCA to that observed in patients taking UDCA^29^ (Extended Data Fig. 9a).

For testing the effects of UDCA against SARS-CoV-2 infection, one hamster was directly inoculated by the intranasal route with 1 × 10^2^ plaque-forming units (PFU) in 100 µl PBS. Each infected hamster was placed on one side of a transmission cage. The cage was divided with an aerated barrier that allowed the infected hamster to be co-housed with previously treated uninfected hamsters housed on the other side, permitting us to study viral infection by aerosol transmission. Daily swabs were collected from all hamsters to monitor the infection by qPCR for the viral N gene. On day 4 after infection, the hamsters were euthanized and lungs and nasal turbinates were collected for quantification of viral infection. The experiment was repeated *n* = 3 times for a total of *n* = 9 UDCA and n = 6 vehicle hamsters. Data were analysed blinded to the identity of the experimental groups.

### Quantification of UDCA concentration

UDCA was quantified from hamster plasma using a liquid chromatography–mass spectrometry (LC–MS) assay that was validated using FDA industry guidelines. Quantification was achieved using LC–MS/MS (6500+ QTRAP, SCIEX) operating in negative mode. UDCA was detected using multiple reaction monitoring (MRM) in which the following ions were monitored for quantification: UDCA (*m*/*z* 391>391 and internal standard mefloquine 379.1>320.1). A stock solution of 1 mg ml^−1^ UDCA was prepared in methanol and stored at 4 °C until use. A standard curve was prepared in plasma by serial dilution from 40,000 ng ml^−1^ to 312.5 ng ml^−1^ and an additional blank solution was also used. Chromatographic separation was achieved using a multi-step gradient with a Acquity BEH C18 column (2.1 mm × 100 mm 1.7 µm; Waters) using mobile phases A (100% water, 0.1% formic acid and 5 mM ammonium formate) and B (90% acetonitrile 10% methanol, 0.1% formic acid and 5 mM ammonium formate). Chromatography was conducted over 3.5 min. At the start of each run, mobile phase A was 80% until 0.5 min, when mobile phase B was increased to 47% over 0.5 min. Mobile phase B was then increased over 1 min to 51%. Mobile phase B was then increased to 100% at 2.5 min, which was held until 3 min. Mobile phase B was reduced to 20% and held until 3.5 min. Samples were extracted from hamster plasma by protein precipitation. In brief, 100 µl of standard, quality control, blank plasma or study sample were treated with 400 µl of acetonitrile. Samples were then vortexed followed by centrifugation at 3,500 rpm for 5 min, and 400 µl of supernatant was transferred to fresh glass vials and evaporated under a steady stream of nitrogen. Samples were reconstituted in 50:50 water methanol and analysed. Inter- and intra- assay variance was assessed by three levels of independent quality controls. The coefficients of variation of accuracy and precision were less than 15% in all assays.

### ESNP of human lungs

For the ex situ perfusion of a single pair of human lungs, two bespoke ESNP circuits (Medtronic) were used. In brief, this circuit facilitates pressure-monitored perfusion with normothermic perfusion solution consisting of bovine serum albumin (BSA) (70 g l^−1^), dextran 40 (5 g l^−1^), modified Krebs Henseleit buffer (9.2 g l^−1^), sodium bicarbonate solution (28 ml l^−1^), calcium chloride (25 ml l^−1^) and heparin (3,750 units per l). The pair of human lungs used was perfused following the physiological principles previously described^59^.

#### Lung ESNP experimental set-up

The experiment shown in Fig. 3 was performed on a pair of lungs that was declined for clinical lung transplantation owing to the donor’s past medical history. The left and the right lungs were divided at the carina and common pulmonary artery bifurcation. For each isolated lung, the pulmonary artery and the pulmonary vein were cannulated and an endobronchial tube was inserted into the main bronchus. The cannulae of each lung were connected to an entirely independent ESNP circuit (control and experimental lung circuits). The endobronchial tube of each lung was connected to an independent mechanical ventilator (Drägerwerk AG & Co. KGaA). Mechanical ventilation was performed using room air with a positive end expiratory pressure of 5 mmHg and a target tidal volume of 130 ml at 5 bpm for the left lung and 140 ml at 6 bpm for the right lung.

#### Timing and duration of ESNP

Perfusion started simultaneously for both lungs. After 30 min of stable perfusion, UDCA or carrier was administered and this time point was defined as 0 h (0 h). ESNP was performed for 6 h for both lungs after administration of UDCA or carrier. The end of experiment time point is defined as 6 h (6 h).

#### Experimental time points and sample collection

Baseline tissue and perfusate samples were collected simultaneously from both lungs before UDCA or carrier administration. We defined these samples as 0-h samples. Corresponding samples were collected simultaneously from each lung at the end of the experiment at 6 h. We defined those as 6-h samples.

The following samples were collected from each lung per time point: *n* = 4 independent lung biopsies, *n* = 4 surgically excised independent samples from the pulmonary artery, *n* = 4 surgically excised independent samples from the main bronchus and *n* = 4 independent samples from the perfusate in each circuit. The independent tissue samples refer to different locations of the organ. Biopsies were surgically excised and the lung parenchymal samples from 0 h were taken from peripheral areas separated from the remaining lung by staples (Medtronic) to seal the defect. The 6-h samples were taken using the same approach. Please also refer to the main-text section ‘FXR regulates infection in human organs’.

### ESNP of donor livers

The OrganOx metra normothermic liver perfusion device was used for ex situ perfusion of human livers as previously described^14,30,32^. The machine, which is clinically used for preservation of livers for transplantation, enables prolonged automated organ preservation by perfusing it with ABO-blood group-matched normothermic oxygenated blood. The perfusion device incorporates online blood gas measurement, as well as software-controlled algorithms to maintain pH, PO2 and PCO2 (within physiological limits), temperature, mean arterial pressure and inferior vena cava pressure within physiological normal limits.

#### Experimental set-up

Two donor livers not used for transplantation were maintained with ESNP. In brief, the hepatic artery, portal vein, inferior vena cava and bile duct were cannulated and connected to the device, and perfusion commenced. One liver was randomly chosen to receive a solution of UDCA dissolved in 0.9% NaCl (experimental liver), whereas the other liver (control) was chosen to receive the same volume of carrier (0.9% NaCl). UDCA was resuspended in 0.9% (w/v) NaCl solution and injected in the blood circuit to achieve a final concentration of 2,000 ng ml^−1^, which is the steady-state concentration of UDCA detected in serum after multiple doses of UDCA^29^.

#### Timing and duration of ESNP

The time of UDCA or carrier administration after the start of ESNP was defined as 0 h (0 h). ESNP was performed for 12 h for both livers after administration of UDCA or carrier. The end of experiment time point is defined as 12 h (12 h).

#### Experimental time points and sample collection

Baseline tissue and perfusate samples were collected from each liver before UDCA or carrier administration. We defined these samples as 0 h samples. Corresponding samples were collected from each liver at the end of the experiment at 12 h. We defined those as 12 h samples.

The following samples were collected from each liver per time point: *n* = 4 independent surgically excised gall bladder samples, *n* = 4 independent liver biopsies and *n* = 4 independent samples from the circulating perfusate. Independent gall bladder samples and liver biopsies were obtained from different locations of the organ. Gall bladder samples were surgically excised and the gall bladder wall was sutured to close the defect.

### Ex vivo infection of human tissue with SARS-CoV-2

The infection of human tissue maintained ex vivo with SARS-CoV-2 occurred in a CL-3 facility after the ESNP experiment. Four independent samples from the lung parenchyma, bronchi and the vasculature (pulmonary artery) of each lung were collected per time point; that is, at the start of the ESNP experiment (0 h) and at the end (6 h) of the lung perfusion. Four independent samples from each ESNP liver gall bladder were collected per time point; that is, at the start of the ESNP experiment (0 h) and at the end (12 h) of the liver perfusion. Sample collection was performed as described in ‘ESNP of donor livers’.

The freshly obtained lung, bronchial, vascular and gall bladder tissues were processed into small rectangular pieces of 0.5 × 0.5 cm and were rinsed with University of Wisconsin solution (lung, bronchial and vascular tissues) or William’s E medium with supplements as previously described^51^ (gall bladder tissue). Washed specimens were placed in wells of a 24-well plate (one specimen per well) and infected with SARS-CoV-2. An inoculum of 1.2 × 10^5^ PFU per ml at 500 µl per well was used. After two hours, the inoculum was removed, and the specimens were washed three times with PBS. The infected human tissue was then cultured in 500 µl of advanced DMEM with supplements^60^ (lung, bronchial and vascular tissues) or William’s E medium with supplements (gall bladder tissue). Supernatant and tissue were collected for qPCR and immunofluorescence at 2 h and 24 h after infection.

### Blood sample collection and processing

Blood samples were collected from patients as part of the UK-PBC Nested Cohort study after obtaining informed consent, anonymized and analysed by a blinded researcher. To obtain serum from full blood, the samples were spun at 4 °C at 1,000*g* for 10 min to allow for serum separation and serum was collected as the supernatant. All blood samples were collected after fasting.

### ACE2 enzymatic activity

ACE2 enzymatic activity was performed on serum samples and tissue lysates using the ACE2 activity fluorometric kit (Abcam ab273297) following the manufacturer’s instructions using SoftMax Pro 5.4.4 on a SpectraMax M2 (Molecular Devices).

### ACE2 measurement in nasal epithelial cells of volunteers

#### Recruitment

Following approval by the local ethics committee (Ethik-Kommission der Ärztekammer Hamburg; ref. no. 2021-300121-WF), the study was advertised in the University Medical Centre Hamburg-Eppendorf amongst clinicians who regularly prescribe UDCA, and are thus familiar with the drug and its possible side effects. Eight clinicians who volunteered to participate in the study were recruited after informed consent. The characteristics of the volunteers are provided in Supplementary Table 5.

#### Study design and exclusion criteria

UDCA was self-administered at the clinically approved dose of 15 mg per kg per day in a single morning dose for 5 days. Nasal epithelium samples for ACE2 measurement were collected each morning before UDCA administration using the Citoswab nasopharyngeal swab collection kit (Corona Smear). Day 0 samples were collected before the first UDCA dose. Daily morning samples were collected during UDCA treatment (days 1–5). After drug washout, repeat nasopharyngeal swabs were collected between days 22 and 28. Volunteers providing samples with no detectable RNA were excluded from the study.

#### ACE2 measurement

RNA was extracted from the Citoswab nasopharyngeal swab collection kit (Corona Smear) using the RNeasy micro kit (Qiagen) according to the manufacturer’s instructions. ACE2 mRNA levels were measured using qPCR (see ‘Immunofluorescence, RNA extraction and qPCR’). The results are shown as fold change over the housekeeping gene *GAPDH*.

### Serum proteome analysis in patients with PBC

Serum proteomic analysis was performed in the UK-PBC patient cohort as described previously^33^. Blood samples were obtained with informed consent and appropriate ethical approval (UK-PBC tissue bioresource, 14/NW/1146). Serum samples were assayed using the Olink proteomics platform (https://olink.com/).

### Patient data

Data for patients with chronic liver disease were collected as described elsewhere^35,61^. In brief, collated results from two open online reporting registries (COVID-Hep.net and SECURE-Liver) were examined. Reports were asked to record cases of laboratory-confirmed COVID-19 in patients with chronic liver disease at the end of the disease course. Anonymous clinical and demographic data were collected and filtered to remove duplicate entries and entries with incomplete records, and to remove data on individuals with prior liver transplantation, who were not over 18 years of age, who were over 90 years of age or who did not have a laboratory-confirmed infection.

Data for patients with liver transplants were collected as described elsewhere^36^. In brief, data from patients with a liver transplant who were alive on 1 March 2020 were examined. Participants who had no COVID-19 infection, were unvaccinated or developed COVID-19 within 30 days of their first UDCA prescription were excluded. The resulting study sample included *n* = 24 patients on UDCA and 95 who were not on UDCA, and this sample was used for the analysis.

### Statistical analyses

Statistical analyses were performed using Microsoft Excel v.16.19, Rstudio (v.1.1.463), GraphPad Prism 9 or Stata 15.1 (StataCorp). The normal distribution of our values was evaluated using the Shapiro-Wilk test where appropriate. For comparison between two groups, a two-tailed Student’s *t*-test or the non-parametric Mann–Whitney test was used depending on the normality of our distribution. To compare matched samples from the same individual, a two-tailed paired Student’s *t*-test was used (the normal distribution of our data was confirmed using the Shapiro-Wilk test). Variance between samples was tested using the Brown-Forsythe test. For comparing multiple groups to a reference group, a one-way ANOVA followed by Dunnett’s test was used between groups with equal variance. Immunofluorescence images are representative of four independent experiments. Data are represented in box plots and elements are defined as follows: centre line, median; box, IQR; whiskers, range; error bars, s.d.

Serum proteomic analysis was performed using Rstudio (v.1.1.463). The correlation between ACE2 levels and UDCA administration was interrogated using multiple linear regression analysis with the lm function in R. ACE2 expression data was defined as the independent variable, and UDCA administration, sex, age, BMI, stage of chronic liver disease according to the Child–Turcotte–Pugh class and alkaline phosphatase were defined as dependent variables.

COVID-Hep and SECURE-Liver data (exploratory cohort): for propensity-score-matched analyses, 1:5 matched samples (using the nearest-neighbour approach) were constructed with hospitalization, physician-reported requirement for intensive care, intensive care admission, mechanical ventilation and death as the outcome variables. The covariables used were age, sex and categorical stage of chronic liver disease according to the Child–Turcotte–Pugh class, diabetes, chronic pulmonary disease (COPD or asthma), immunosuppressive therapy, increased BMI (BMI > 25), and the presence of non-alcoholic fatty liver disease, owing to its association with increased COVID-19 risk. Notably, ARLD is also associated with increased COVID-19 risk; however, patients could not be propensity-score-matched between the two cohorts, as there were no patients with ARLD receiving UDCA. Consequently, patients with ARLD were excluded.

VOCAL data (validation cohort): for propensity-score-matched analyses, 1:3 matched samples (using a Greedy matching algorithm) were constructed with moderate, severe or critical COVID-19 and severe or critical COVID-19 according to the NIH severity scale as the outcome variables. The covariables used were age, sex, ethnicity, location within the United States, diabetes, BMI, COPD, type of immunosuppressive therapy (calcineurin inhibitor with or without anti-metabolite therapy) and dominant SARS-CoV-2 variant.

Propensity-score matching was performed using the teffects function in Stata. The average treatment effect on the treated (ATET) was calculated with robust Abadie-Imbens standard errors and derived 95% CIs are presented.

### Reporting summary

Further information on research design is available in the Nature Portfolio Reporting Summary linked to this article.

## Online content

Any methods, additional references, Nature Portfolio reporting summaries, source data, extended data, supplementary information, acknowledgements, peer review information; details of author contributions and competing interests; and statements of data and code availability are available at 10.1038/s41586-022-05594-0.

## Supplementary information

Supplementary Information This file contains Supplementary Fig. 1 (Gating strategy for flow cytometry analyses) and Supplementary Tables 1–5 and 7–9 (see separate Excel file for Supplementary Table 6).

Reporting Summary

Supplementary Table 6 UK-PBC cohort characteristics relative to Extended Data Fig. 10.

Supplementary Data This file contains code for Extended Data Fig. 10.

## Data Availability

scRNA-seq data are available on ArrayExpress under accession number E-MTAB-8495. Source data are provided with this paper.

## Source data

Source Data Fig. 2
Source Data Extended Data Fig. 7
Source Data Extended Data Fig. 8
