# Supplementary material for: FXR inhibition may protect from SARS-CoV-2 infection by reducing ACE2
Source: Nature. 2022 Dec 5;615(7950):134–42. doi: 10.1038/s41586-022-05594-0 (PMC9977684; doi:10.1038/s41586-022-05594-0)
Supplement: Supplementary file 1 — This file contains Supplementary Fig. 1 (Gating strategy for flow cytometry analyses) and Supplementary Tables 1–5 and 7–9 (see separate Excel file for Supplementary Table 6). [file 41586_2022_5594_MOESM1_ESM.pdf]

nature portfolio

https://doi.org/10.1038/s41586-022-05594-0

Supplementary information

FXR inhibition may protect from SARS-CoV-2 infection by reducing ACE2

In the format provided by the
authors and unedited

Supplementary Information

FXR inhibition may protect from SARS-CoV-2 infection by reducing ACE2

Teresa Brevini*, Mailis Maes, Gwilym J. Webb, Binu V. John Claudia D. Fuchs, Gustav Buescher, Lu Wang, Chelsea Griffiths, Marnie Brown, William Scott, Pehuén Pereyra Gerber, William T. H. Gelson, Stephanie Brown, Scott Dillon, Daniele Muraro, Jo Sharp, Megan Neary, Helen Box, Lee Tatham, James Stewart, Paul Curley, Henry Pertinez, Sally Forrest, Petra Mlcochova, Sagar S. Varankar, Mahnaz Darvish-Damavandi, Victoria L. Mulcahy, Rhoda E. Kuc, Thomas L. Williams, James Heslop, Davide Rossetti, Vasileios Galanakis, Marta Vila-Gonzalez, Olivia C. Tysoe, Thomas W. M. Crozier, Johannes Bargehr, Sanjay Sinha, Sara S. Upponi, Corinna Fear, Lisa Swift, Kourosh Saeb-Parsy, Susan E. Davies, Axel Wester, Hannes Hagström, Espen Melum, Darran Clements, Peter Humphreys, Jo Herriott, Edyta Kijak, Helen Cox, Chloe Bramwell, Anthony Valentijn, Christopher J. R. Illingworth, Bassam Dahman, Dustin R. Bastaich, Raphaella D. Ferreira, Thomas Marjot, Eleanor Barnes, Andrew M. Moon, A. Sidney Barritt IV, Ravindra K. Gupta, Stephen Baker, Anthony P. Davenport, Gareth Corbett, Vassillis G. Gorgoulis, Simon J. A. Buczacki, Joo-Hyeon Lee, Nicholas J. Matheson, Michael Trauner, Andrew J. Fisher, Paul Gibbs, Andrew J. Butler, Christopher J. E. Watson, George F. Mells, Gordon Dougan, Andrew Owen, Ansgar W. Lohse, Ludovic Vallier*† and Fotios Sampaziotis*†

† These authors share senior authorship.

*Correspondence to: Fotios Sampaziotis, fs347@cam.ac.uk; Ludovic Vallier, lv255@cam.ac.uk; Teresa Brevini, tb647@cam.ac.uk.

Supplementary Figure S1. Gating strategy for flow cytometry analyses.

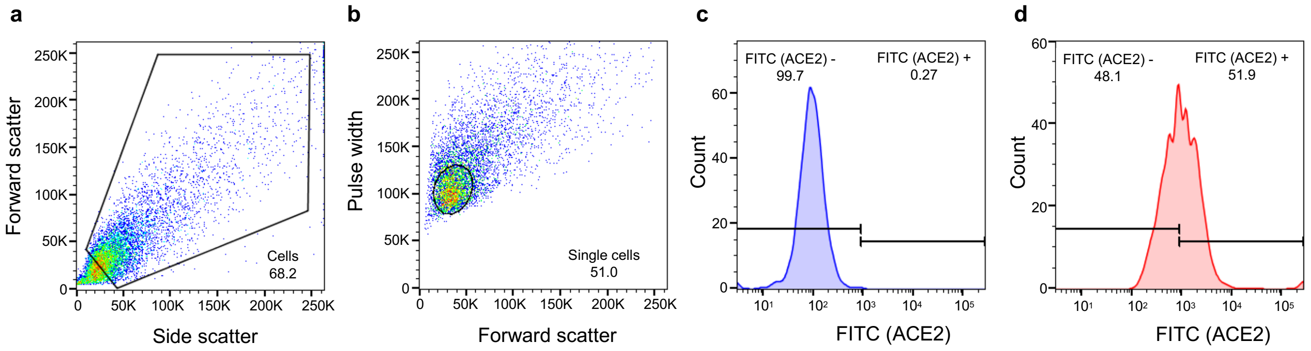

Supplementary Figure S1. Gating strategy for flow cytometry analyses. (**a-d**) Representative flow cytometry plots showing gating strategy for all flow cytometric analyses, gating on: (**a**) exclusion of debris; (**b**) exclusion of doublets; (**c**) secondary-only control to exclude negative population; (**d**) representative ACE2+ population.

Supplementary Table S1. List of primary and secondary antibodies.

Target protein	Company	Product code	Dilution
ACE2	R&D	AF933	1:50 / 1:100
ACE2	abcam	ab15348	1:500
ACE2	abcam	ab108209	1:500 / 1:100
EPCAM	R&D	MAB9601	1:50 / 1:100
EPCAM	R&D	AF960	1:100
Cytokeratin 19	abcam	ab7754	1:100
Cytokeratin 19	abcam	ab52625	1:100
SOX2	abcam	ab15830	1:100
SOX2	R&D	AF2018	1:100
NKX2.1	abcam	ab72876	1:100
Cytokeratin 5	Thermo Fisher	MA5-17057	1:100
Surfactant protein C	Merck Millipore	AB3786	1:300
Acetylated alpha tubulin	Sigma	T7451	1:500
CD31	Novus biological	NB100-2284	1:100
CD31	abcam	ab119339	1:100
Alpha smooth muscle actin	abcam	ab124964	1:100
SARS-CoV spike glycoprotein	abcam	ab273433	1:100
SARS-CoV-2 nucleocapsid	Sino Biological	40143-R019	1:100
SOX17	R&D	AF1924	1:100
FXR	Novus biological	NBP2-16550	1:100
FXR	Santa Cruz	sc-25309 X	1:100
Actin	abcam	ab208080	1:100
Alexa Fluor Donkey Anti-Rabbit 568	Invitrogen	A10042	1:1000
Alexa Fluor Donkey Anti-Rabbit 488	Invitrogen	A21206	1:1000
Alexa Fluor Donkey Anti-Rabbit 568	Invitrogen	A10042	1:1000
Alexa Fluor Donkey Anti-Goat 488	Invitrogen	A11055	1:1000
Alexa Fluor Donkey Anti-Goat 568	Invitrogen	A11057	1:1000
Alexa Fluor Donkey Anti-Mouse 488	Invitrogen	A21202	1:1000
Alexa Fluor Donkey Anti-Mouse 647	Invitrogen	A31571	1:1000

Supplementary Table S2. List of primers used for QPCR.

Target gene	Primer sequence (5' – 3')	
HMBS	Forward	GGAGCCATGTCTGGTAACGG
	Reverse	CCACGCGAATCACTCTCATCT
GAPDH	Forward	AGGACTCATGACCACAGTCCATGC
	Reverse	GATGACCTTGCCCACAGCCTT
KRT19	Forward	ACGACCATCCAGGACCTGCGG
	Reverse	TCCCACTTGGCCCCTCAGCGTA
ACE2	Forward	CTCCTAACCAGCCCCCTGTT
	Reverse	TGGAGGCATAAGGATTTTCTCCAC
SARS-CoV-2 RdRp	Forward	ATGGGTTGGGATTATCCTAAATGTGA
	Reverse	GCAGTTGTGGCATCTCCTGATGAG
MS2	Forward	TGGCACTACCCCTCTCCGTATTC
	Reverse	GTACGGGCGACCCCACGATGAC
NRB02	Forward	CCTGCCTGAAAGGGACCATCC
	Reverse	GCACCAGGGTTCCAGGACTTC
NR1H4	Forward	GCTTTGCTGAAAGGGTCTGC
	Reverse	CAGAATGCCCAGACGGAAGT
IL-1β	Forward	AAACAGATGAAGTGCTCCTTCCAGG
	Reverse	TGGAGAACACCACTTGTTGCTCCA
IL-6	Forward	AATTCGGTACATCCTCGACGG
	Reverse	GGTTGTTTTCTGCCAGTGCC
IFNα	Forward	GACTCCATCTTGGCTGTGA
	Reverse	TGATTTCTGCTCTGACAACCT
IFNλ	Forward	TCGCTTCTGCTGAAGGACTGCA
	Reverse	CCTCCAGAACCTTCAGCGTCAG
KRT7	Forward	GATTGCTGGCCTTCGGGGT
	Reverse	TCATCACAGAGATATTCACGGCTC
CFTR	Forward	AGTTGCAGATGAGGTTGGGC
	Reverse	AAAGAGCTTCACCCTGTCGG
GGT1	Forward	GTGAGAGCAGTTGGCTGTGC
	Reverse	GTTGAACTCTGCTGTGGGGC
SOX17	Forward	CGCACGGAATTTGAACAGTA
	Reverse	GGATCAGGGACCTGTCACAC
mGAPDH	Forward	GCACAGTCAAGGCCGAGAAT
	Reverse	GCCTTCTCCATGGTGGTGAA
mACE2	Forward	TCCATTGGTCTTCTGCCATCC
	Reverse	AACGATCTCCCGCTTCATCTC
mSHP	Forward	AAGGGCTTGCTGGACAGTTA
	Reverse	TCTCTTCTTCCGCCCTATCA
hGAPDH	Forward	AGGTTGTCTCCTGCGACTTCA
	Reverse	GCATCAAAGGTGGAAGAGTGG
hACE2	Forward	AAAGTGGTGGGAGATGAAGCGA
	Reverse	GAACAGAGCTGCAGGGTCAC

KRT19, CK19; *NRB02*, SHP; *NR1H4*, FXR; *KRT7*, CK7.

Supplementary Table S3. List of primers used for ChIP-QPCR.

Target gene	Primer sequence (5' – 3')	
OSTα	Forward	AGTTCAGGGCTTTGGGTAATTAAAC
	Reverse	GGTGGAGGTCAGGGAAGGAAGA
ACE2	Forward	CGCTATCTTGAGGAAGAAGGGGAA
	Reverse	AGCAGGTACAAAGCATATGCAACC
ACE2 negative region	Forward	AAGCGAGCTCAGTGTCCTCA
	Reverse	AGGTAGGCCCTTGAACCCTG

Supplementary Table S4. List of primers used for luciferase reporter.

Target	Primer sequence (5' – 3')	
gACE2	Forward	AGTTCAGGGCTTTGGGTAATTAAAC
	Reverse	GGTGGAGGTCAGGGAAGGAAGA
gSHP	Forward	ATTGAGATGCAGATCGCAGATCTCGAGCCCCTCATGGTTAGGGATCTGCTCTCAC
	Reverse	TAGGTACCGAGCTCTTACGCGTGCTAGCCCCCTGCTTCTGGCTGACAACAG
IR1 mutagenesis	Forward	AAGCGAGCTCAGTGTCCTCA
	Reverse	AGGTAGGCCCTTGAACCCTG

Supplementary Table S5. Volunteer cohort characteristics relative to Fig. 4b.

Age	Sex	Weight (kg)	Daily UDCA dose (mg)
61	Male	80	1250
42	Male	82	1250
47	Female	50	750
34	*Female*	*68*	*1000*
46	*Male*	*74*	*1250*
30	Male	63	1000
28	Female	61	1000
45	Female	75	1000

Participants shown in italics were excluded due to undetectable RNA levels. See methods.

Supplementary Table S6. UK-PBC cohort characteristics relative to Extended Data Figure 10.

Please, refer to downloadable xls file and Supplementary File S1 for the code employed in the analysis.

Supplementary Table S7. COVID-Hep and SECURE-Liver registries patient cohort characteristics.

	Total	No UDCA	UDCA	Propensity score matched (5:1) (No UDCA:UDCA)
**n, total**	788	757	31	155
**Sex (Female)**	347 (44.0%)	319 (42.1%)	28 (90.3%)	139 (89.6%)
**Age (years; median, IQR)**	58 (47–67)	58 (47–67)	56 (44–63)	57 (47–63)
**BMI (overweight)**	445 (56.4%)	434 (57.3%)	11 (35.4%)	44 (28.3%)
**Diabetes**	295 (37.4%)	291 (38.4%)	4 (12.9%)	24 (15.4%)
**NAFLD**	378 (47.9%)	376 (49.6%)	2 (6.4%)	10 (6.4%)
**Immunosuppression**	78 (9.9%)	70 (9.25%)	8 (25.8%)	41 (26.4%)
**Chronic respiratory disease**	104 (13.2%)	101 (13.3%)	3 (9.6%)	20 (12.9%)
Liver disease severity				
**CLD, no cirrhosis**	447 (56.7%)	423 (55.8%)	24 (77.4%)	122 (78.7%)
**CTP A cirrhosis**	184 (23.3%)	182 (24.0%)	2 (6.4%)	9 (5.8%)
**CTP B cirrhosis**	101 (12.8%)	97 (12.8%)	4 (12.9%)	19 (12.2%)
**CTP C cirrhosis**	56 (7.1%)	55 (7.2%)	1 (3.2%)	5 (3.2%)
COVID-19 outcome				
**Hospitalised**	636 (80.7%)	625 (82.5%)	11 (35.4%)	91 (58.7%)
**ICU requirement**	214 (27.1%)	213 (28.1%)	1 (3.2%)	21(13.5%)
**ICU admission**	172 (21.8%)	171 (22.5%)	1 (3.2%)	20 (12.9%)
**Invasive ventilation**	122 (15.4%)	121 (15.9%)	1 (3.2%)	15 (9.6%)
**Death**	117 (14.8%)	117 (15.4%)	0 (0%)	14 (9.0%)

UDCA, ursodeoxycholic acid; IQR, interquartile range; BMI, body mass index; CLD, chronic liver disease; CTP, *Child-Turcotte-Pugh* class; BMI, body mass index; NAFLD, non-alcoholic fatty liver disease; ICU, intensive care unit.

Supplementary Table S8. Liver disease aetiology relative to Fig. 4d.

Liver disease aetiology	No UDCA	UDCA
**Cholestatic only**	**20 (12.9%)**	**20 (64.5%)**
Primary biliary cholangitis (PBC)	1 (0.6%)	20 (64.5%)
Primary sclerosing cholangitis (PSC	17 (11.0%)	0 (0.0%)
Other cholestatic (ICP, Biliary atresia)	2 (1.3%)	0 (0.0%)
**Autoimmune**	**47 (30.3%)**	**9 (29.0%)**
Autoimmune hepatitis (AIH)	46 (29.7%)	1 (3.2%)
Overlap syndrome (PBC/AIH)	1 (0.6%)	8 (25.8%)
**Non-alcoholic fatty liver disease (NAFLD)**	**10 (6.5%)**	**2 (6.5%)**
NAFLD	8 (5.2%)	0 (0.0%)
NAFLD and PBC	0 (0.0%)	2 (6.5%)
NAFLD and viral hepatitis	2 (1.3%)	0 (0.0%)
**Viral hepatitis**	**63 (40.6%)**	**0 (0.0%)**
**Other (congenital; cryptogenic; veno-occlusive disease; unknown)**	**15 (9.7%)**	**0 (0.0%)**
**n, total**	155	31

Supplementary Table S9. VOCAL registry patient cohort characteristics.

	Total	No UDCA	UDCA	Propensity score matched (3:1) (No UDCA:UDCA)
**n, total**	119	95	24	72
**Sex (Female)**	3 (2.5%)	1 (1.1%)	2 (8.3%)	1 (1.4%)
**Age (years; median, IQR)**	58.9	59.0 (9.2)	58.5 (9.0)	57.6 (8.9)
**BMI (BMI; median IQR)**	29.75	30.0 (4.3)	28.8 (7.9)	29.6 (4.1)
**Diabetes**	100 (84.0%)	78 (82.1%)	22 (91.7%)	61 (84.7%)
**Hypertension**	97 (81.5%)	77 (81.1%)	20 (83.3%)	60 (83.3%)
**Kidney transplantation**	21 (17.6%)	17 (17.9%)	4 (16.7%)	16 (22.2%)
**Smoker**	67 (56.3%)	56 (59.0%)	11 (45.8%)	45 (62.5%)
Immunosuppression				
**Calcineurin inhibitors**	109 (91.6%)	87 (91.6%)	22 (91.7%)	68 (94.4%)
**Anti-metabolites**	73 (61.3%)	61 (64.2%)	12 (50.0%)	46 (63.9%)
**Chronic respiratory disease**	44 (37.0%)	37 (39.0%)	7 (29.2%)	27 (37.5%)
Ethnicity				
**White**	74 (62.2%)	59 (62.1%)	15 (62.5%)	44 (61.1%)
**Black**	20 (16.8%)	14 (14.7%)	6 (25.0%)	12 (16.7%)
**Hispanic/Latino**	8 (6.7%)	8 (8.4%)	0 (0%)	5 (6.9%)
**Other**	16 (13.4%)	14 (14.7%)	2 (8.3%)	11 (15.3%)
**Unknown**	1 (0.8%)	0 (0%)	1 (4.2%)	0 (0%)
Location in the United States				
**Northeast**	28 (23.5%)	25 (26.3%)	3 (12.5%)	28 (25.0%)
Southeast	20 (16.8%)	17 (17.9%)	3 (12.5%)	12 (16.7%)
**Midwest**	34 (28.6%)	21 (22.1%)	13 (54.2%)	20 (27.8%)
**South**	18 (15.1%)	15 (15.8%)	3 (12.5%)	13 (18.1%)
**Northwest**	6 (5.0%)	6 (6.3%)	0 (0%)	0 (0%)
**Southwest**	13 (10.9%)	11 (11.6%)	2 (8.3%)	9 (12.5%)

UDCA, ursodeoxycholic acid; IQR, interquartile range; BMI, body mass index.
