# Supplementary material for: FXR inhibition may protect from SARS-CoV-2 infection by reducing ACE2
Source: Nature. 2022 Dec 5;615(7950):134–42. doi: 10.1038/s41586-022-05594-0 (PMC9977684; doi:10.1038/s41586-022-05594-0)
Supplement: Supplementary file 2 — Reporting Summary [file 41586_2022_5594_MOESM2_ESM.pdf]

nature research

Corresponding author(s): Fotios Sampaziotis, Ludovic Vallier, Teresa Brevini

Last updated by author(s): October 16, 2022

Reporting Summary

Nature Research wishes to improve the reproducibility of the work that we publish. This form provides structure for consistency and transparency in reporting. For further information on Nature Research policies, see our Editorial Policies and the Editorial Policy Checklist.

Statistics

For all statistical analyses, confirm that the following items are present in the figure legend, table legend, main text, or Methods section.

n/a	Confirmed	
☐	☒	The exact sample size (*n*) for each experimental group/condition, given as a discrete number and unit of measurement
☐	☒	A statement on whether measurements were taken from distinct samples or whether the same sample was measured repeatedly
☐	☒	The statistical test(s) used AND whether they are one- or two-sided<br>*Only common tests should be described solely by name; describe more complex techniques in the Methods section.*
☐	☒	A description of all covariates tested
☐	☒	A description of any assumptions or corrections, such as tests of normality and adjustment for multiple comparisons
☐	☒	A full description of the statistical parameters including central tendency (e.g. means) or other basic estimates (e.g. regression coefficient) AND variation (e.g. standard deviation) or associated estimates of uncertainty (e.g. confidence intervals)
☐	☒	For null hypothesis testing, the test statistic (e.g. F, t, r) with confidence intervals, effect sizes, degrees of freedom and P value noted<br>*Give P values as exact values whenever suitable.*
☒	☐	For Bayesian analysis, information on the choice of priors and Markov chain Monte Carlo settings
☒	☐	For hierarchical and complex designs, identification of the appropriate level for tests and full reporting of outcomes
☒	☐	Estimates of effect sizes (e.g. Cohen's *d*, Pearson's *r*), indicating how they were calculated

Our web collection on statistics for biologists contains articles on many of the points above.

Software and code

Policy information about availability of computer code

Data collection	BD FACS Diva 8.0.3 (BD Bioscience; for analyses on LSR-II); ZEN 2011 SP7 (Zeiss) on a Zeiss LSM 700 or 710 confocal microscope; QuantStudio 5 384 Well Block (Thermo Fisher); SoftMax Pro 5.4.4 on SpectraMax M2 (Molecular Devices); HT7800 TEM operating software version 01.21 (Hitachi) on a HT7800 transmission electron microscope (Hitachi High Technologies, Japan).
Data analysis	Microsoft Excel v 16.19; GraphPad Prism 9; Stata 15.1 (StataCorp, College Station, TX, USA); FlowJo v 10; ImageJ 2.0.0-rc-69/1.53f; Anaconda-Navigator 1.9.12; Jupyter Notebook 6.0.3; Rstudio (version 1.1.463).

For manuscripts utilizing custom algorithms or software that are central to the research but not yet described in published literature, software must be made available to editors and reviewers. We strongly encourage code deposition in a community repository (e.g. GitHub). See the Nature Research guidelines for submitting code & software for further information.

Data

Policy information about availability of data

All manuscripts must include a data availability statement. This statement should provide the following information, where applicable:

- Accession codes, unique identifiers, or web links for publicly available datasets
- A list of figures that have associated raw data
- A description of any restrictions on data availability

Single-cell RNA sequencing data are available on ArrayExpress. Accession number: E-MTAB-8495. Source data are provided with this paper.

Field-specific reporting

Please select the one below that is the best fit for your research. If you are not sure, read the appropriate sections before making your selection.

☒ Life sciences ☐ Behavioural & social sciences ☐ Ecological, evolutionary & environmental sciences

For a reference copy of the document with all sections, see nature.com/documents/nr-reporting-summary-flat.pdf

Life sciences study design

All studies must disclose on these points even when the disclosure is negative.

Sample size

No statistical analyses were performed to predetermine sample size. The samples size for each experiment is specified in the corresponding figure legend. At least 4 biological replicates were used for in vitro experiments. At least 4 mice and 3 hamsters per group were used for in vivo experiments. For ESNP experiments 4 independent samples from the same organ before and after UDCA treatment were used for each tissue. For animal experiments, group sizes were estimated based on previous study variance and cage capacity. For in vitro and ex vivo experiments, sample size of n=4 was chosen as the minimum number of samples required to adequately provide standard deviation, median and range. For human prospective study, the maximum number of individuals that could be recruited during the study period was used. For retrospective clinical studies, the maximum number of individuals in our cohort meeting the study criteria was used.

Data exclusions

In the COVID-Hep.net and SECURE-Liver registries data were filtered to remove duplicate entries, those with incomplete records, those with prior liver transplantation, those younger than 18 years of age and older than 90 years of age, and those without laboratory confirmed infection. Due to the absence of patients with Alcohol Related Liver Disease (ARLD) in the UDCA group, patients with ARLD were excluded from the analysis in both groups. In the UK-PBC proteomic study data were filtered to remove duplicate entries, those with incomplete records and those classified as non-responders to treatment according to PARIS2 criteria. For the study involving participants from the University Medical Centre Hamburg-Eppendorf, two individuals were excluded because of undetectable RNA levels in nasopharyngeal swabs. No further data were excluded from the analyses presented in this manuscript. In the VOCAL cohort of liver transplant recipients, participants who had no COVID-19 infection, were unvaccinated or developed COVID-19 within 30 days from their first UDCA prescription were excluded.

Replication

All experiments were repeated and validated as stated in the respective figure legends.

Randomization

No formal randomization method was used to assign primary organoids to study groups. For in vitro experiments primary organoids were plated in 24-well plates and different wells of the same plates were randomly allocated to one of the 4 experimental groups (Carrier only; CDCA; CDCA+UDCA; CDCA+ZGG). For in vivo experiments, animals were randomly allocated to the treatment or control group. For analysis of the UK-PBC proteomic cohort covariables such as age, sex, BMI, stage of liver disease (Child-Turcotte-Pugh class) and ALP were controlled for via multiple linear regression. For analysis of the COVID-Hep.net and SECURE-Liver registries patient cohort covariables such as age, sex, diabetes, NAFLD and stage of liver disease (Child-Turcotte-Pugh class) were controlled for via propensity score matching analyses. For the VOCAL cohort of liver transplant recipients covariable such as age, sex, ethnicity, location within the United States, diabetes, BMI, COPD, type of immunosuppressive therapy (calcineurin Inhibitor with or without anti-metabolite therapy) and dominant SARS-CoV-2 variant were controlled for via propensity score matching analyses.

Blinding

Blinding was performed for all experiments. If blinding was not possible at the time of the experiment, specifically in directly inoculated hamster samples and organs perfused ex situ, samples were collected and anonymised and sample processing was subsequently performed by a blinded researcher.

Reporting for specific materials, systems and methods

We require information from authors about some types of materials, experimental systems and methods used in many studies. Here, indicate whether each material, system or method listed is relevant to your study. If you are not sure if a list item applies to your research, read the appropriate section before selecting a response.

Materials & experimental systems

n/a	Involved in the study	
☐	☒	Antibodies
☐	☒	Eukaryotic cell lines
☒	☐	Palaeontology and archaeology
☐	☒	Animals and other organisms
☐	☒	Human research participants
☒	☐	Clinical data
☒	☐	Dual use research of concern

Methods

n/a	Involved in the study	
☒	☐	ChIP-seq
☐	☒	Flow cytometry
☒	☐	MRI-based neuroimaging

Antibodies

Antibodies used

A full list of the antibodies used can be found in Supplementary Table S1.

Validation

Anti-ACE2; R&D; AF933; dilution 1:50 / 1:100; https://resources.rndsystems.com/pdfs/datasheets/af933.pdf?v=20221029&_ga=2.68548246.663064109.1667061858-1717995244.1667061858.
Anti-ACE2; abcam; ab15348; dilution 1:500; https://www.abcam.com/ace2-antibody-ab15348.html.
Anti-ACE2; abcam; ab108209; dilution 1:500 / 1:100; https://www.abcam.com/ace2-antibody-epr4436-ab108209.html.
Anti-EPCAM; R&D; MAB9601; dilution 1:50 / 1:100; https://resources.rndsystems.com/pdfs/datasheets/mab9601.pdf?v=20221029&_ga=2.232101272.663064109.1667061858-1717995244.1667061858.
Anti-EPCAM; R&D; AF960; dilution 1:100; https://resources.rndsystems.com/pdfs/datasheets/af960.pdf?v=20221029&_ga=2.232101272.663064109.1667061858-1717995244.1667061858.
Anti-Cytokeratin 19; abcam; ab7754; dilution 1:100; https://www.abcam.com/cytokeratin-19-antibody-a53-ba2-cytoskeleton-marker-ab7754.html.
Anti-Cytokeratin 19; abcam; ab52625; dilution 1:100; https://www.abcam.com/cytokeratin-19-antibody-ep1580y-cytoskeleton-marker-ab52625.html.
Anti-SOX2; abcam; ab15830; dilution 1:100; https://www.abcam.com/sox2-antibody-ab15830.html.
Anti-SOX2; R&D; AF2018; dilution 1:100; https://resources.rndsystems.com/pdfs/datasheets/af2018.pdf?v=20221029&_ga=2.31979961.663064109.1667061858-1717995244.1667061858.
Anti-NKX2.1; abcam; ab72876; dilution 1:100; https://www.abcam.com/ttf1-antibody-8g7g31-ab72876.html.
Anti-Cytokeratin 5; Thermo Fisher; MA5-17057; dilution 1:100; https://www.thermofisher.com/antibody/product/Cytokeratin-5-Antibody-clone-2C2-Monoclonal/MA5-17057.
Anti-Surfactant protein C; Merck Millipore; AB3786; dilution 1:300; https://www.merckmillipore.com/GB/en/product/Anti-Prosurfactant-Protein-C-proSP-C-Antibody,MM_NF-AB3786.
Anti-Acetylated alpha tubulin; Sigma; T7451; dilution 1:500; https://www.sigmaaldrich.com/GB/en/product/sigma/t7451.
Anti-CD31; Novus biological; NB100-2284; dilution 1:100; https://www.novusbio.com/products/cd31-pecam-1-antibody_nb100-2284.
Anti-CD31; abcam; ab119339; dilution 1:100; https://www.abcam.com/cd31-antibody-hec7-ab119339.html.
Anti-Alpha smooth muscle actin; abcam; ab124964; dilution 1:100; https://www.abcam.com/alpha-smooth-muscle-actin-antibody-epr5368-ab124964.html.
Anti-SARS-CoV spike glycoprotein; abcam; ab273433; dilution 1:100; https://www.abcam.com/sars-spike-glycoprotein-antibody-1a9-ab273433.html.
Anti-SARS-CoV-2 nucleocapsid; Sino Biological; 40143-R019; dilution 1:100; https://www.sinobiological.com/antibodies/cov-nucleocapsid-40143-r019.
Anti-SOX17; R&D; AF1924; dilution 1:100; https://resources.rndsystems.com/pdfs/datasheets/af1924.pdf?v=20221029&_ga=2.206978708.663064109.1667061858-1717995244.1667061858.
Anti-FXR; Novus biological; NBP2-16550; dilution 1:100; https://www.novusbio.com/products/fxr-nr1h4-antibody_nbp2-16550.
Anti-FXR; Santa Cruz; sc-25309 X; dilution 1:100; https://datasheets.scbt.com/sc-25309.pdf.
Anti-Actin; abcam; ab208080; dilution 1:100; https://www.abcam.com/alexa-fluor-555-actin-antibody-epr16769-ab208080.html.

Eukaryotic cell lines

Policy information about cell lines

Cell line source(s)

Vero E6 cells (ATCC™ CRL – 1586) were kindly donated by Gordon Dougan's laboratory. HEK293 cells (ATCC™ CRL – 1573) and HEK293T cells (ATCC™ CRL – 3216) were kindly donated by Nicholas J Mathenson's laboratory. Primary human tissues were used to derive organoids. All human tissues were obtained with full ethical approval (REC reference numbers: 12/EE/0253, NRES Committee East of England, Cambridge Central and 15/EE/0152 NRES Committee East of England, Cambridge South) and informed consent from the patients or the donors' families.

Authentication

None of the cell lines used were authenticated

Mycoplasma contamination

All line tested negative for mycoplasma.

Commonly misidentified lines
(See ICLAC register)

No commonly misidentified cell lines were used in the study.

Animals and other organisms

Policy information about studies involving animals; ARRIVE guidelines recommended for reporting animal research

Laboratory animals

FVB/N female mice aged between 9 and 12 weeks were used. Golden Syrian Hamsters were purchased from Janvier Labs (France). Age matched male hamsters weighing between 80 – 100g were used. All animals were housed in a 12 hours/12 hours dark/light cycle, with a humidity of 45-65% and temperature of 20-24°C.

Wild animals

No wild animals were used in the study.

Field-collected samples

No field collected samples were used in the study.

Ethics oversight

The mouse study was approved by the Animal Ethics Committee of the Medical University of Vienna and the Federal Ministry of Science, Research and Economy (BMWFW-66.009/0008-WF/3b/2015) and was performed according to the Animal Research: Reporting of In Vivo Experiments (ARRIVE) guidelines.
The hamster study was performed in accordance with the UK Home Office Animals Scientific Procedures Act (ASPA, 1986). Additionally, all studies were approved by the University of Liverpool Animal Welfare and Ethical Review Board and performed under UK Home Office licences PP9284915 and PP4715265.

Note that full information on the approval of the study protocol must also be provided in the manuscript.

Human research participants

Policy information about studies involving human research participants

Population characteristics	Population characteristics of the human research participants are listed in Supplementary Table S5, Supplementary Table S6, Supplementary Table S7 and Supplementary Table S8.
Recruitment	Existing samples or datasets were used (e.g., data from COVID-Hep.net and SECURE-Liver registries, serum samples from the UK-PBC Cohort study, data from the VOCAL cohort). For the study involving volunteers from the University Medical Centre Hamburg-Eppendorf, following approval by local ethics committee (Ethik-Kommission der Ärztekammer Hamburg; Ref.No. 2021-300121-WF), the study was advertised in the University Medical Centre Hamburg-Eppendorf amongst clinicians regularly prescribing UDCA, and thus familiar with the drug and its possible side-effects. 8 clinicians who volunteered to participate in the study were recruited following informed consent. We appreciate the potential for selection and confounding bias in any study which is not a clinical trial, and this limitation is clearly stated in the results and discussion sections.
Ethics oversight	The COVID-Hep.net and SECURE-Liver registries data were deemed not to constitute human research by Clinical Trials and Research Governance at the University of Oxford (https://covid-hep.net/img/CTRG_COVID-Hep_20200402.pdf) and by the Institutional Review Board of University of North Carolina (https://covidcirrhosis.web.unc.edu/faq/) respectively. The study involving volunteers from the University Medical Centre Hamburg-Eppendorf was performed with informed consent and ethical approval from the Ethik-Kommission der Ärztekammer Hamburg (Ref.No. 2021-300121-WF). The study involving patients with PBC from the UK-PBC Nested Cohort was performed with informed consent and ethical approval from the National Research Ethics Committee (NREC) North West (14/NW/1146). The study involving patients from the VOCAL cohort was performed with informed consent and ethical approval from the Miami VA Institutional Review Board (Unique study approval ID 1477437-22).

Note that full information on the approval of the study protocol must also be provided in the manuscript.

Flow Cytometry

Plots

Confirm that:

☒ The axis labels state the marker and fluorochrome used (e.g. CD4-FITC).

☒ The axis scales are clearly visible. Include numbers along axes only for bottom left plot of group (a 'group' is an analysis of identical markers).

☒ All plots are contour plots with outliers or pseudocolor plots.

☒ A numerical value for number of cells or percentage (with statistics) is provided.

Methodology

Sample preparation	Cholangiocyte organoids obtained from intrahepatic ducts, common bile duct and gallbladder human tissues obtained from biopsies, deceased transplant organ donors or liver explants after obtaining informed consent were used. Organoids were harvested from Matrigel using Cell Recovery Solution for 20 minutes in ice. Organoids were treated with StemPro Accutase for 5 minutes at 37°C to dissociate cell clumps into single cells; fixed in 4% PFA for 20 minutes at 4°C; blocked with 10% donkey serum (Gibco) + 0.1% Triton-X in PBS (Gibco) for 30 minutes; stained with primary antibodies from abcam (See supplementary table S1) in 1% doney serum (Gibco) + 0.1% Triton X in PBS (Gibco) for 1 hour at room temperature; primary antibody was subsequently washed in PBS (Gibco) for 5 minutes for three times; organoids were then stained with secondary antibodies (See supplementary table S1) for 1 hour at room temperature and filtered through a 40-µm filter and analysed. A detailed description of the protocol can be found in Methods section "Flow cytometry analyses".
Instrument	BD LSR-II from BD Biosciences.
Software	FlowJo version 10.
Cell population abundance	No post-sort fraction was collected. For each experiment at least 10.000 events were captured.
Gating strategy	Initial cell populations were gated using FSC and SSC to remove cell debris and large cell clumps. Subsequently, FSC-W and FSC-A were used to gate only single cells and remove doublets. This population was then used in fluorescent histograms. Samples stained for the secondary antibody only were used to set the gates. The gating strategy employed to analyse flow cytometry data can be found in Supplementary Figure S1.

☒ Tick this box to confirm that a figure exemplifying the gating strategy is provided in the Supplementary Information.
