# Supplementary material for: FXR inhibition may protect from SARS-CoV-2 infection by reducing ACE2
Source: Nature. 2022 Dec 5;615(7950):134–42. doi: 10.1038/s41586-022-05594-0 (PMC9977684; doi:10.1038/s41586-022-05594-0)
Supplement: Supplementary file 4 — This file contains code for Extended Data Fig. 10. [file 41586_2022_5594_MOESM4_ESM.docx]

**Supplementary File S1.** Code for Extended Data Figure 10

# https://www.blopig.com/blog/2019/06/a-brief-introduction-to-ggpairs/

library("tidyverse")

library("ggpubr")

library("cowplot")

library("GGally")

library("jtools")

source('utils.R')

data <- read.csv("./DATA.csv")

data <- data %>% filter(!is.na(ACE2) & !is.na(Sex) & !is.na(Age) &!is.na(BMI) & !is.na(ALP) & !is.na(ChildPughScore))

data$DRUG <- factor(data$DRUG, levels = c("Cohort B", "Cohort A"))

data$ALP <- factor(data$ALP, levels = c("NORMAL", "CHOLESTATIC"))

regress.out <- lm(data$ACE2 ~ data$DRUG + data$ChildPughScore + data$Sex+ data$BMI + data$Age + data$ALP)

summary(regress.out)

multiple.regression.with.ci(regress.out)
